# Visual exploration dynamics are low-dimensional and driven by intrinsic factors

**DOI:** 10.1038/s42003-021-02608-x

**Published:** 2021-09-17

**Authors:** Andrea Zangrossi, Giorgia Cona, Miriam Celli, Marco Zorzi, Maurizio Corbetta

**Affiliations:** 1 Department of Neuroscience, University of Padova, Padova, Italy; 2 Padova Neuroscience Center (PNC), University of Padova, Padova, Italy; 3 Venetian Institute of Molecular Medicine, VIMM, Padova, Italy; 4 Department of General Psychology, University of Padova, Padova, Italy; 5 IRCCS San Camillo Hospital, Venice, Italy

**Keywords:** Attention, Saccades

## Abstract

When looking at visual images, the eyes move to the most salient and behaviourally relevant objects. Saliency and semantic information significantly explain where people look. Less is known about the spatiotemporal properties of eye movements (i.e., how people look). We show that three latent variables explain 60% of eye movement dynamics of more than a hundred observers looking at hundreds of different natural images. The first component explaining 30% of variability loads on fixation duration, and it does not relate to image saliency or semantics; it approximates a power-law distribution of gaze steps, an intrinsic dynamic measure, and identifies observers with two viewing styles: static and dynamic. Notably, these viewing styles were also identified when observers look at a blank screen. These results support the importance of endogenous processes such as intrinsic dynamics to explain eye movement spatiotemporal properties.

## Abstract (web summary)

Andrea Zangrossi et al. analyze individual variability in eye movements when healthy participants view images of natural scenes. Their results suggest that eye movement patterns can be summarized by three principle components, and depend more on subjects’ intrinsic characteristics than the visual contents of the scene.

## Introduction

The exploration of visual scenes through eye movements is a complex behaviour mediated by the sequential and recursive interactions of multiple cognitive processes. For many years it was thought that eye movements were predominantly guided by stimulus-driven factors such as the sensory distinctiveness of different objects in the visual field. A highly influential model by Itti and colleagues^1^ proposed a neural network that selects attended locations in a single topographical saliency map formed by multiscale low-level features of different objects. Indeed, the pattern of eye movements while viewing complex scenes is in part predicted by the saliency of the visual images (e.g., videos^2^ or pictures^3^). A cognitive model for the control of visual attention during the search is also based on the parallel analysis of visual features^4^.

However, since the seminal studies of Yarbus^5^, it has been known that the patterns of eye movements depend not only on low-level features, but also on the behavioural relevance of stimuli in the visual scene, e.g., people, faces, etc., as well as the goals of the observer. Therefore, current theories, and computational models, propose that visual exploration is guided both by sensory and cognitive signals^3,6,7^. However, even the best models of fixation location to static natural images account for about 30% of the potential information present in an image^8^. In general, these models see the brain as a sensory-motor analyzer whose activity is mainly driven by the analysis and transformation of sensory stimuli into motor decisions.

Understanding naturalistic eye movement behaviour is not limited only to the topographical aspects of visual exploration, but also to its spatiotemporal dynamics. Most studies to date have focused on the spatial pattern of fixations during natural visual exploration^1–8^. The spatiotemporal pattern of fixation selection is of increasing interest to the field, but much less studied. One study found that eye movement parameters (e.g., fixation duration, saccade amplitude) were correlated across different laboratory tasks (e.g., sustained fixation vs. search vs. Stroop paradigm), and that the majority of variability across subjects could be summarized with a single factor putatively related to visual attention^9^. Other studies found important individual variability, but also high consistency of spatiotemporal patterns across tasks (e.g., visual search vs. fixation^10–13^) or different versions of the same picture^14^.

These results suggest that eye movement spatiotemporal patterns may reflect an intrinsic or endogenous signature relatively independent of visual input or goal^10^. These patterns have been related to individual cognitive styles^15^, personality traits^16^, and genetic influence^17^.

Here we aimed to quantify the role of stimulus-driven vs. endogenous (intrinsic) parameters by examining eye movement spatiotemporal features in a large group of healthy participants while they viewed a large set of real-world scenes vs. when they viewed a blank screen, i.e., without any visual stimulus.

First, we were interested in testing whether the variability of eye movement (e.g., amplitude, velocity), fixation (e.g., duration, rate), and pupil parameters across many subjects, and across many visual scenes, was explained with a relatively high or low number of dimensions. High dimensionality would indicate that different subjects look differently, or that different image features yield different eye movement patterns or both. A low dimensional solution, instead, would indicate that eye movement/fixation patterns across subjects can be explained with a few components relatively independent of stimulus content.

Secondly, we asked whether eye movement spatiotemporal features during visual exploration were modulated by sensory or semantic content—stimulus-driven information— or by a power-law-like distribution of gaze steps^18^—a measure of intrinsic dynamics. Power law relations are ubiquitously found in nature and predict many complex phenomena such as earthquakes^19^, volcanic eruptions^20^, stock market^21^, and foraging behaviour of many species^22,23^. Power-law behaviour in biological systems is thought to reflect the intrinsic constraints of the system, e.g., anatomical connections or neural dynamics in the case of the brain^24–27^. Power-law scaling relations have been also found in eye movement patterns during visual search^18^.

Finally, to further tease apart sensory-driven vs. endogenous factors, we measured spatiotemporal eye movement parameters in the absence of visual stimuli, i.e., when looking at a blank screen, and compare them to parameters found during visual exploration. A large parameter difference in the two conditions would be consistent with the importance of sensory-driven factors; in contrast, their similarity would be more consistent with intrinsic factors controlling eye movement patterns.

This study highlights a low dimensionality of eye movements spatiotemporal dynamics and a role of intrinsic factors (i.e., similarity between eye movements while watching scenes vs. blank screen, and power-law distribution of eye-movements). These results suggest that visual exploration dynamics are partially independent from the visual content.

## Results

**Fig. 1:**
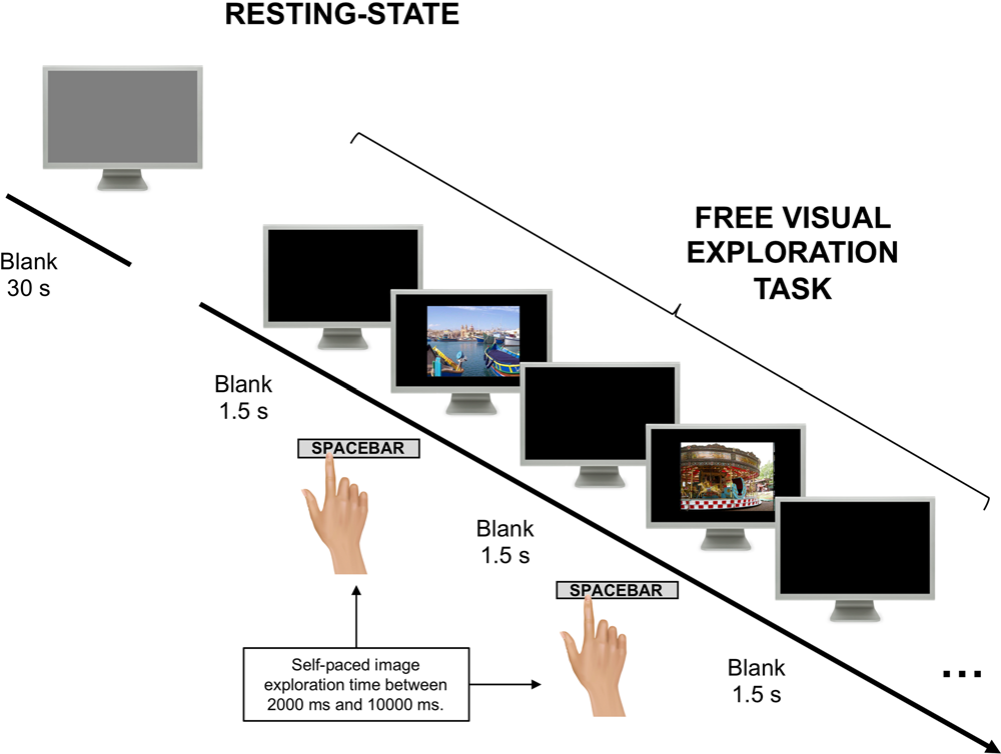
Experimental paradigm. The experiment begins with a blank screen viewing condition in which participants were asked to look at a grey screen for 30 s. Next, participants were presented with a set of static images representing a variety of different scenes (e.g., indoor, outdoor scenes with or without humans or natural vs. man-made). Subjects were asked to explore with their eyes the picture and press a bar when ready to explore the next picture. They were also told that they will be asked some questions at the end of the experiment.

Healthy participants (*n* = 120) were recruited at the University of Padova, with *n* = 114 satisfying inclusion criteria (Supplementary Table 1 for demographic information). All participants had normal or corrected-to-normal (i.e., glasses, *n* = 54) vision. Participants (aged 19–34 years) were tested in a single experimental session lasting approximately 2 h during which their eye-movements were tracked while watching a blank screen or freely exploring a set of 185 real-world scenes. These scenes were selected from a larger set of 36,500 pictures^28^ (see Supplementary Fig. 1 for the flowchart used for selection) to be representative of the following categories: indoor vs. outdoor, which in turn were divided into natural vs. man-made. The content of the pictures had no emotional valence and half of them contained human figures (Supplementary Fig. 2 shows exemplars of each category). Participants were asked to look at each picture carefully, as they were told that they would be asked some questions later on, and, when ready, to advance to the next picture by pressing the spacebar on the computer keyboard (Fig. 1). After the free viewing phase, they were asked to recall and describe a subset of images which were repeated five times. The average number of freely recalled details was 59.97 (SD = 20.5; range: 22–141; 2.6% false memories) across all the five images.

A large set of eye movement features (i.e., 58) were extracted including: fixation duration and number, gaze step length and the number of direction flips, pupil diameter, velocity, exploration time, etc. (Supplementary Table 2). A battery of behavioural tests and questionnaires was then administered to evaluate working memory, visuospatial memory, impulsivity, anxiety, and personality traits (see Supplementary Table 1 for a list of the measures). All volunteers received 10€ for their participation.

### Low dimensionality in eye movement dynamics

**Fig. 2:**
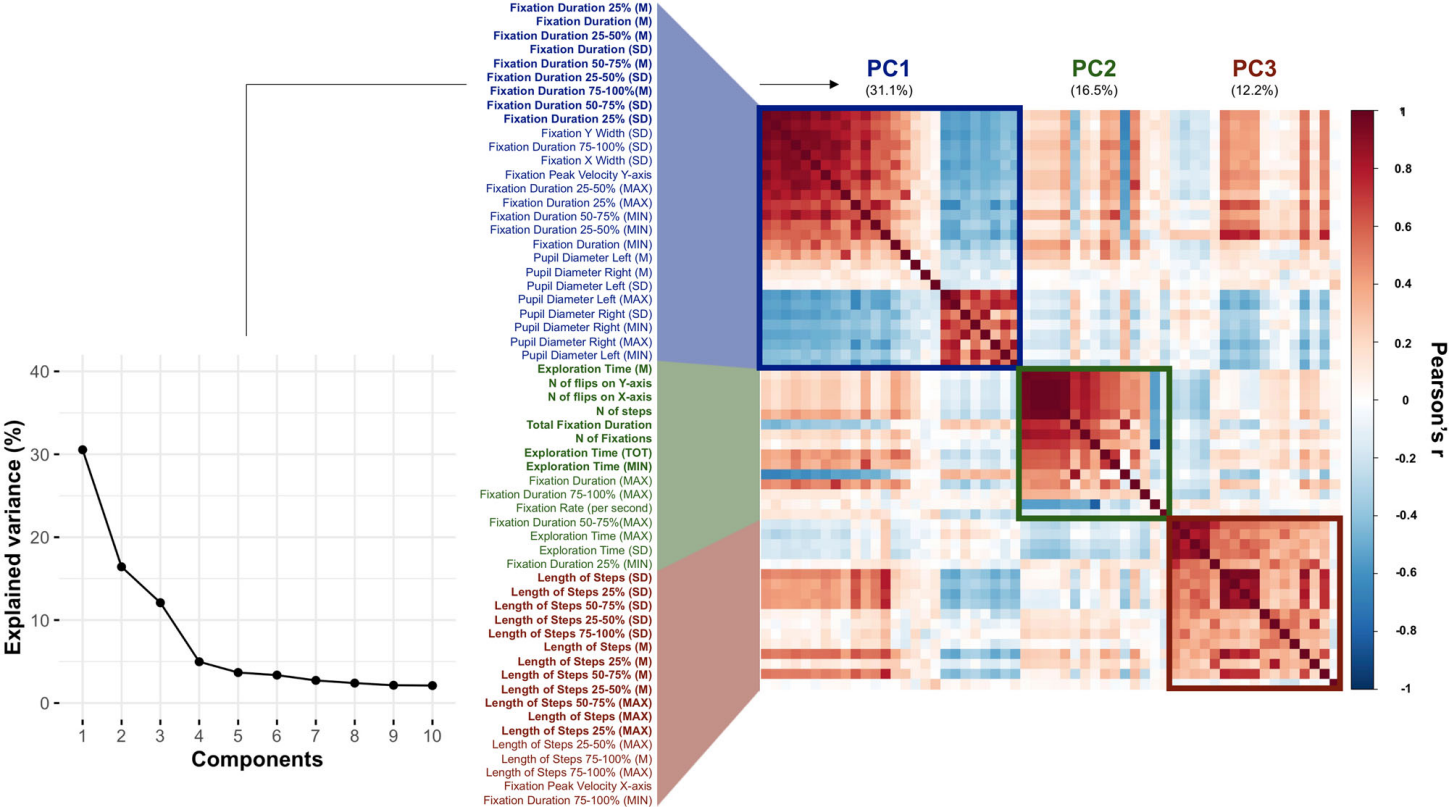
Correlation matrix of spatiotemporal features and principal components (PCs). The line plot shows the variance explained by different PCs. The matrix shows the correlation (Pearson’s *r*) between features, which are ordered according to their loadings in the first three PCs. The colour of *Y*-axis labels indicates the PC with the highest loading for the corresponding feature, and features written in bold are those with loadings > 0.2.

The first question we addressed is whether eye movement dynamical features during visual exploration are ‘different’ or ‘similar’ across individual observers and many different images. We examined the pattern of correlation across eye movement features, images, and subjects by running a principal component analysis (PCA) on the scaled and mean-centred full set of features extracted from the gaze data acquired during the exploration of images. A three-components solution accounted for 59% of the total variance (Fig. 2 and Supplementary Table 3). The first component (30.5%) mainly loaded on fixation duration, the second (16.4%) on gaze step direction and exploration time, and the third (12.1%) on gaze step length, where gaze steps are defined as gaze shifts between consecutive timepoints (see “Methods” section for further details). Pupil diameter inversely correlated with fixation duration, in line with previous literature showing that pupils tend to contract during prolonged fixations^29^, and suggesting the existence of a common mechanism that regulates both pupillary response and fixation disengagement through the activity of the superior colliculus, as demonstrated in primates’ brain^30,31^.

**Fig. 3:**
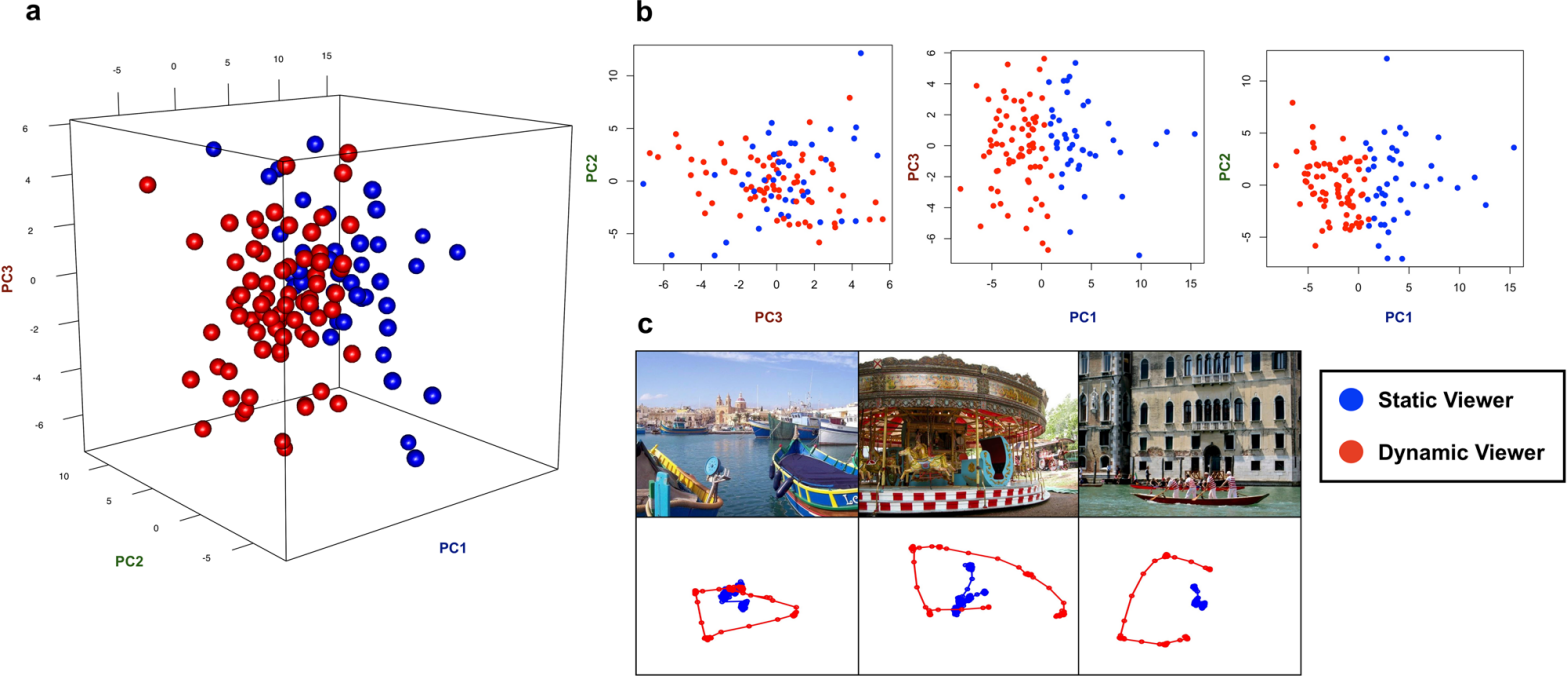
Relation between clusters and principal components. **a** Clusters’ projection in the three-dimensional space defined by the first three principal components. **b** Two-dimensional relation between PC scores. The values of PC1 are those best describing the two clusters; **c** Examples of Static and Dynamic eye-movements pattern (each dot represents gaze position sampled at a timepoint). Static viewers are represented in blue and Dynamic viewers in red.

We then performed a *k*-means cluster analysis splitting the sample into two clusters. The *k* = 2 clustering solution emerged as the most reliable after comparing the similarity between *k*-means and hierarchical clustering solutions obtained with different distance measures and values of k (see Supplementary Fig. 2 for details). Figure 3a shows the distribution of observers along the first three principal component (PC) scores. The best separation (ROC analysis accuracy = 99.9%, 95% C.I. [95.83–100] with cut-off value of 0.69, AUC = 99.9%) was obtained along the PC1 score (Fig. 3b). Participants with high PC1 scores were nicknamed "Static Viewers" because they showed a lower fixation rate but longer fixations. Participants with low PC1 scores were nicknamed "Dynamic Viewers" because they showed more frequent but shorter fixations (Fig. 3c).

**Fig. 4:**
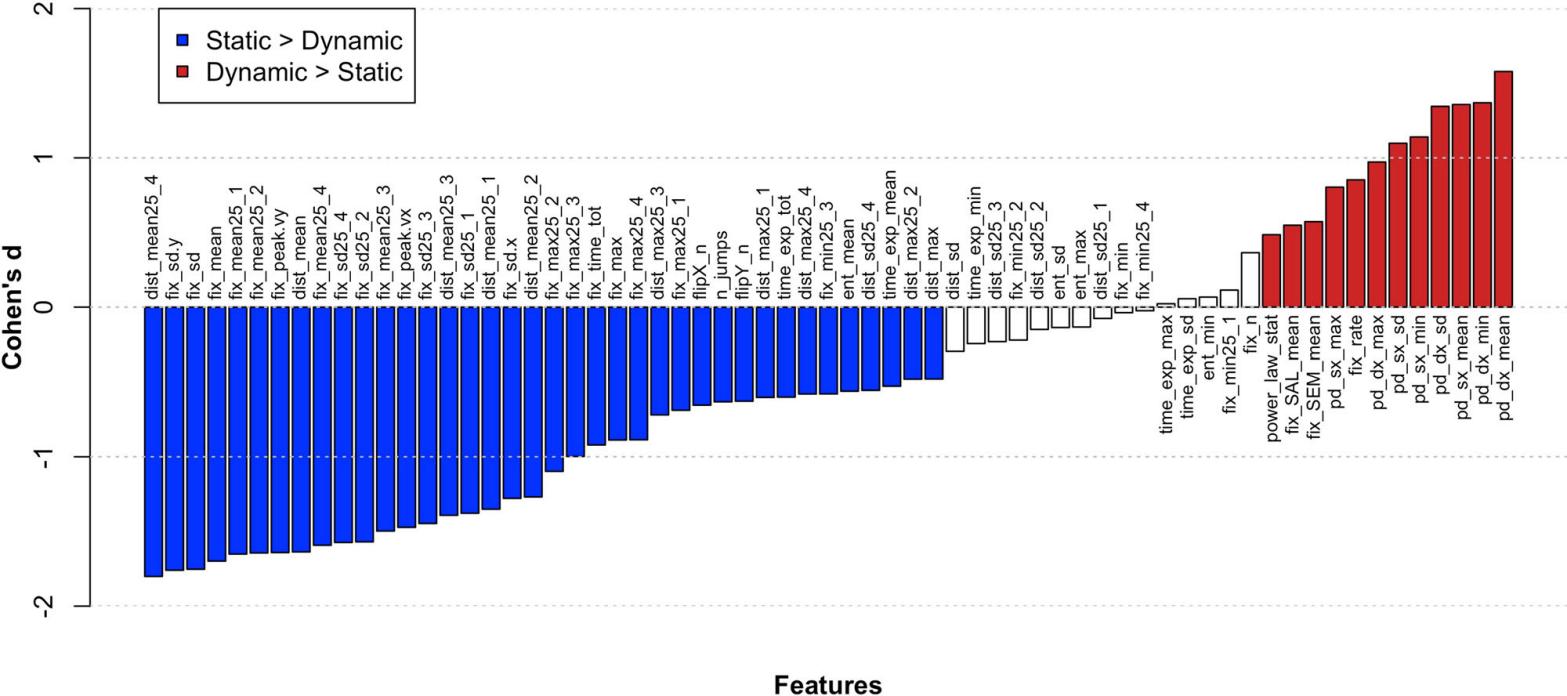
Characterization of the static vs. dynamic viewing styles. A series of t-test was run comparing Static (*n* = 42) and Dynamic (*n* = 72) Viewers across all features. Supplementary Table 2 shows a description of features’ labels. In order for the different metrics to be comparable, an effect-size measure (i.e., Cohen’s d) has been computed (*Y*-axis). Significant results surviving false discovery rate (FDR) correction for multiple comparisons are represented by coloured bars. Red bars indicate a significantly higher value for Dynamic viewers compared to Static viewers in the corresponding feature, while blue bars reveal the opposite pattern (i.e., Static viewers higher than Dynamic). White bars indicate t-tests not reaching significance after FDR correction.

In general, static viewers explored images for a longer time and showed on average higher amplitude and more numerous gaze steps, more gaze flips, smaller pupil diameter, as well as a distribution of gaze steps more similar to a power law. Moreover, they looked less at regions in the image with high semantic and saliency information (see “Methods” section for details on the extraction of semantic and saliency information). Dynamic viewers showed an opposite pattern of features, with a higher fixation rate, more fixations, higher pupil diameter, and a distribution of gaze steps less similar to a power law. Figure 4 shows a characterization of the viewing styles in terms of individual features and their relative effect size (Cohen d).

The robustness of this solution was tested by splitting the images in odd and even, computing a PCA in each subset, and correlating the corresponding PC1 scores. We found a high degree of similarity (for all images vs. odd; all images vs. even; and even vs. odd images, all *r* values > 0.97, Supplementary Fig. 4). Furthermore, each participant’s cluster label remained substantially the same when the cluster analysis was run on even (92.1%, i.e., 105/114) or odd (97.4%, i.e., 111/114) images.

To check that the component scores (PC1-3) were a good model of the original observations, we used PC1, PC2, and PC3 scores to reconstruct the original features matrix and compared the similarity of the resulting reconstruction (Supplementary Fig. 5A). As expected, the most accurate reconstruction was obtained using PC1, compared to the other components. Next, we reconstructed individual patterns of features for exemplar Dynamic and Static viewers. The PC1-reconstructed patterns showed the highest similarity with the original ones, measured with the Pearson’s correlation coefficient (Supplementary Fig. 5B; Static viewer *r* = 0.86; Dynamic viewer *r* = 0.77).

**Fig. 5:**
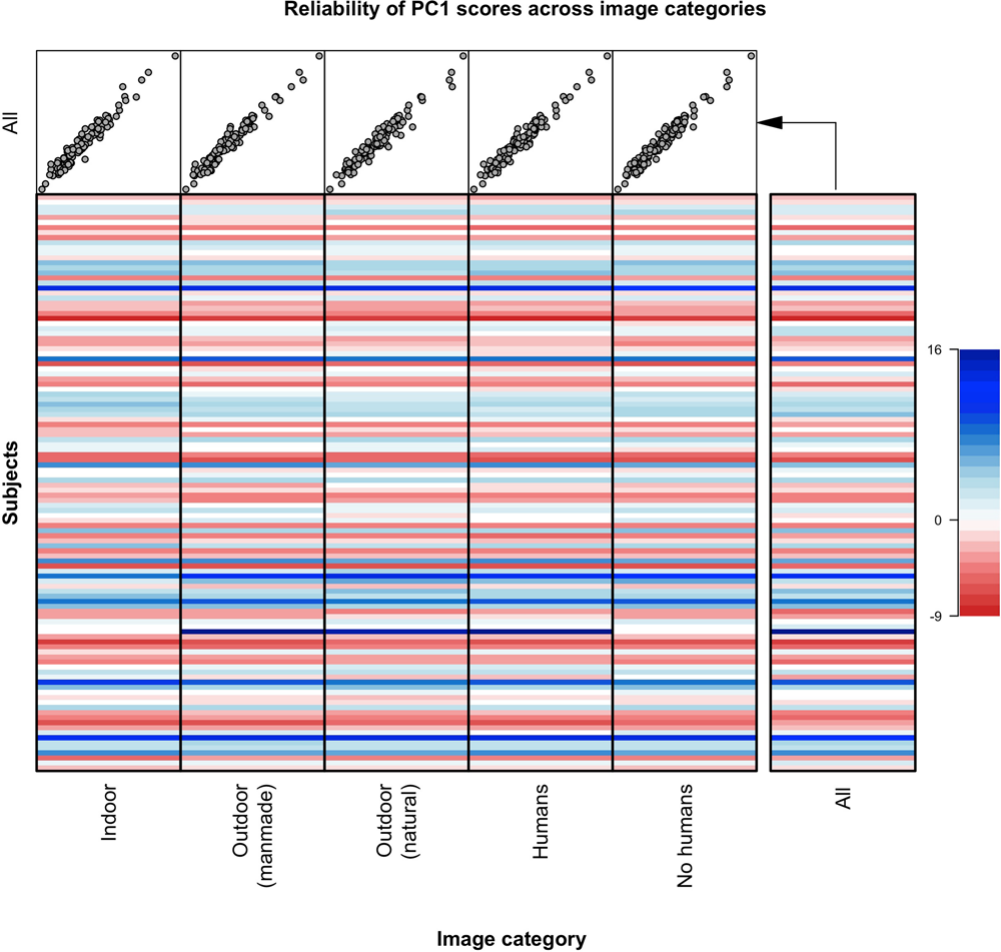
Reliability of the first principal component (PC1) across image categories. The full set of features used for the principal component analysis (PCA) in the main analysis was extracted separately for each image category (i.e., indoor, outdoor natural, outdoor manmade, scenes with humans, scenes without humans). Next, we computed category-specific individual PC1 scores in the component space of the main PCA by applying PC1 loadings (calculated on all images) on features computed from each category. This procedure compares PC1 scores obtained on all images vs. specific image category. The upper five scatterplots represent the correlation between PC1 scores extracted from each specific image category (*x*-axis) and from all images (*y*-axis). The similarity is very high (all Pearson’s *r* = 0.97). The matrix shows the PC1 scores for each subject across different image categories. Note high variability across subjects and similarity across image categories.

Next, we examined if PC1 scores were modulated across different image-categories. We first computed the set of features for each image category (i.e., indoor, outdoor natural, outdoor manmade, scenes with humans, scenes without humans), separately. Then, we computed individual PC1 scores from category-specific features by applying PC1 loadings calculated on all images. This procedure allows to obtain comparable individual scores within the same components space. PC1 scores obtained from all images were very similar to those obtained from different category-specific features (all Pearson’s *r* = 0.97; Fig. 5). Inspection of the PC1 scores at the individual subject level shows that subjects with high/low PC scores overall maintained high/low PC scores for each different image category.

Overall, these findings support a low dimensionality of eye movement dynamic features across many subjects and types of visual scene, and the idea that eye movement dynamic features are relatively independent of image content.

### Relative influence of sensory, semantic, and endogenous variables on eye movement dynamics

Once established that eye movement dynamics across many subjects and visual images can be summarized with a low number of components, we examined more directly if eye movement across subjects was predicted by stimulus-driven or intrinsic factors. We used PC1 scores as dependent variable in a linear regression model that included for each subject and across images: (1) the mean of local sensory saliency values across fixations (SAL), computed as an estimate of the overlap between fixation positions and the local values of the salience-based map derived for each scene from the Itti and Koch model^1^; (2) the mean of local semantic values (SEM), computed similarly on semantic maps for each scene derived from a convolutional neural network trained on 10,000,000 images^28^ (see “Methods” sections for further details, and Supplementary Fig. 6 for a graphical representation of the procedure used to compute SAL and SEM variables); (3) the complexity of visual exploration topography quantified with Shannon Entropy (ShEn), a measure of visual search strategy^32,33^; (4) the Kolmogorov−Smirnov distance (KSD) between the individual distribution of gaze steps and a power-law distribution. Since power laws in a neural system suggest the existence of intrinsic system constraints^24^, we used this measure to study the intrinsic component of eye movements dynamics, as previously suggested^34,35^.

**Fig. 6:**
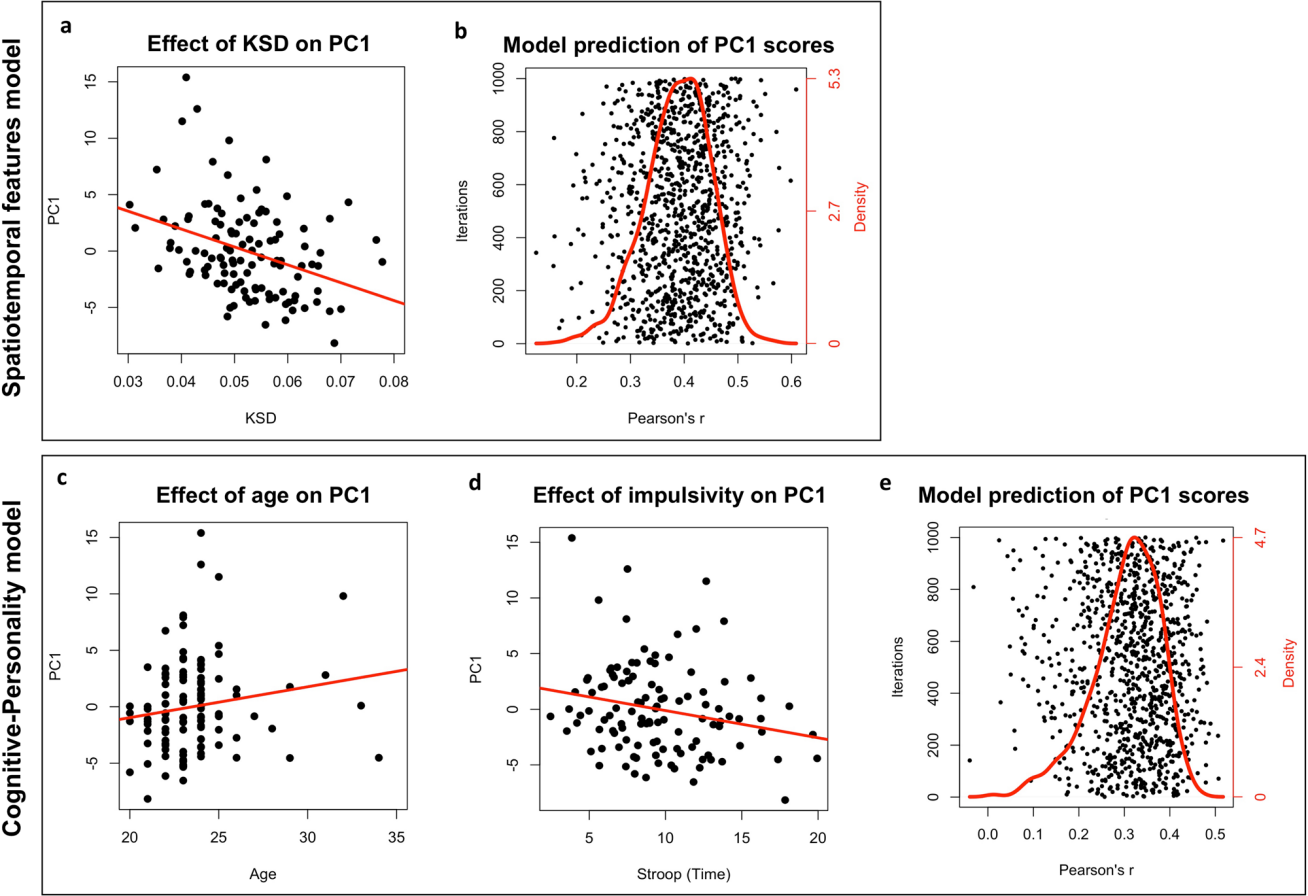
Significant results of regression models and prediction performance. **a** Significant relation between KSD and PC1 scores in the best visual exploration model (i.e., *Spatiotemporal features model*); **b** Pearson’s correlation values between actual and model-predicted PC1 scores obtained over 1,000 iterations of split-half validation procedure. At each iteration the sample (*n* = 114) was randomly split into two halves, one was used as training set to fit the regression model and the other one (i.e., test set) was used to assess the model prediction of PC1 scores for unseen data. The red line indicates the frequency distribution of the correlation values in the scatter plot. The peak of the red line indicates the mean *r* value = 0.42. Significant effects of Age (**c**) and Stroop test (**d**) on PC1 scores in the Cognitive-Personality model; **e** Pearson’s correlation values between actual and model-predicted PC1 scores as described before (see **b**). The peak of the red line indicates the mean *r* value = 0.32.

Four nested linear regression models were built and compared by means of likelihood ratio test (LRT) to determine whether adding a predictor significantly improved model fit. All models included PC1 scores as dependent variable and a different set of predictors. Specifically, M1 included only SAL; M2 included SAL and SEM, M3 included SAL, SEM, and ShEn, and M4 included SAL, SEM, ShEn, and KSD. The LRT showed that M4 was significantly the best model (*F*[1, 109] = 14.49, *p* < .001; see Supplementary Table 4 upper part for details) and led to an increase in the explained variance of about 10% (i.e., *R*^2^_M1_ = 0.083, *R*^2^_M2_ = 0.087, *R*^2^_M3_ = 0.099, *R*^2^_M4_ = 0.198; all values indicate adjusted *R*^2^). This model (i.e., including all predictors; model *F*[4, 109] = 7.59, *p* < .001) showed a significant effect of KSD (*t* = −3.79, *p* < .001; Fig. 6a and Supplementary Fig. 7) and a trend to significance for ShEn (*t* = 1.76, *p* = 0.081). In contrast, SEM and SAL were not significant even though the set of pictures was highly variable in terms of semantic and saliency content (Supplementary Fig. 6). See *Spatiotemporal features model* in Supplementary Table 5 for further details. These results suggest that the model best explaining a significant fraction of PC1 scores (~20% variance explained) is the one containing KSD, and that SAL and SEM do not significantly contribute to explain spatiotemporal patterns of eye movements during free viewing of static natural images.

### Control analyses on PC1−PC3

The M4 model was validated in a split-half design in which 57/114 participants were randomly selected to fit the model parameters while the remaining 57 were used only for testing (i.e., prediction of PC1 scores). This procedure was repeated 1,000 times and the Pearson’s *r* coefficient was collected for each iteration to test the correlation between actual and predicted PC1 scores. All correlations were positive (97.4% of them were significant), with a mean Pearson’s *r* value of 0.42 (SD = 0.078; Fig. 6b).

Next, to rule out the possibility that the results were biased by the eye-tracker’s relatively low spatial resolution (~0.2°, 120 Hz acquisition rate), we checked the similarity of eye-movements patterns to power-laws, as computed through the KSD, using different thresholds of gaze-step length (0.2°–8.1°). Specifically, we removed gaze-steps smaller than each threshold, recomputed the KSD calculation, and the linear regression model predicting PC1 values. This analysis showed that the contribution of KSD was stable across multiple thresholds (0.2°, 0.4°, 0.8°, 1.6°, 3.2°, 4.0°, and 4.9°) eliminating the possibility that this effect was driven by small eye-movements not detected by the eye-tracker (Supplementary Fig. 8).

In control analyses, we ran the same model on PC2 (loading on gaze steps direction flips and exploration time) and PC3 (loading on gaze steps length). The full model (SAL, SEM, ShEn, KSD) indicated that KSD was predictive of PC2 (*t* = −2.96 *p* = 0.004), while SEM was predictive of PC3 (*t* = −2.45 *p* = 0.02). Again, we did not find a significant contribution of the SAL variable.

This analysis shows that the pattern of eye movement dynamic features during visual exploration of scenes is explained by a few components (~60% variance across images and subjects). These components can be used to separate two styles of viewing (>90% accuracy of classification) that are not predicted by sensory salience. On the other hand, the visual exploration style was significantly predicted (~20% variance) by intrinsic dynamics captured by the similarity of the eye gaze steps length distribution to a power law.

### Relative influence of sensory, semantic, and endogenous variables on fixations distribution

Given most of the literature and available models focus on the spatial pattern of fixations^1–8^, we investigated whether intrinsic factors (distance between the distribution of gaze step length and a power law as measured by KSD) significantly contributed to the spatial distribution of fixations. However, since KSD is usually computed across all eye movements (fixation) in an image, we developed a method to estimate KSD from the distribution of gaze steps in different locations of an image. Maps of regional KSD values were then compared to corresponding fixation density maps (FDMs), saliency, and semantic maps (See “Methods” section and Supplementary Fig. 9 for details). A mixed-effects model approach was chosen since it allows to simultaneously take into consideration not only factors under experimental control (i.e., fixed effects), but also the so-called random effects (e.g., repeated measures). In our case, we built three mixed-effects models (MM) including a random intercept for both images and subjects, and the following fixed effects: saliency (MM1), saliency and semantic (MM2), saliency, semantic, and KSD (MM3). ShEn was not included due to its high correlation with FDM. The different models were compared by means of a LRT. Specifically, we tested if the addition of KSD to a model including saliency and semantic information explained the topographical distribution of fixations more accurately. The model comparison was run across subjects and also within each subject independently. In the latter case, MMs were built with random intercept only for images.

The model including saliency, semantic, and KSD (MM3) was the best model (χ²[1] = 681.12, *p* < .001; Supplementary table 4 lower part). This was confirmed at the individual level with MM3 being the best model in >70% of subjects. The winning model showed strong significant effects of SAL (*t* = 167.16, *p* < .001), SEM (*t* = 123.32, *p* < .001), and KSD (*t* = −26.11, *p* < .001; Fig. 6a and Supplementary Fig. 7). See *Fixation topography model* in Supplementary Table 5 for further details.

These results confirm the role of saliency and semantic information in explaining the topography of fixations (i.e., FDMs), and suggest a significant contribution of KSD, despite the proportion of explained variance did not increase significantly (i.e., *R*^2^_MM1_ = 0.151, *R*^2^_MM2_ = 0.195, *R*^2^_MM3_ = 0.196).

### Eye movement dynamics during blank screen viewing

Given the significant influence of intrinsic eye movement dynamics on visual exploration, we asked whether the pattern of eye movements could be used to accurately classify participants during visual exploration of a blank screen (herein blank screen viewing). A positive result would strongly support the idea that intrinsic factors independent of visual analysis are important in controlling eye movement patterns. To test this hypothesis, we applied the same pipeline of analysis, i.e., features extraction and PCA to 30 s of blank screen viewing data prior to the presentation of the first image. It should be emphasized that subjects had not seen any of the images prior to the blank screen viewing observation period. Fourteen participants were removed from this analysis because they maintained steady fixation at the centre of the screen. The blank screen viewing data analysis was thus conducted on a sample of *n* = 100 subjects. The results did not change when all subjects were included.

The PCA on blank screen viewing data (Supplementary Table 3 and Supplementary Fig. 10) showed also a low dimensionality with three components explaining ~50% of the variance (23.4, 19, and 8.4%, respectively). Not surprisingly, the order of components during blank screen viewing was not the same as during visual exploration. Fixation features that loaded on PC1 during visual exploration moved to a weak PC3 during blank screen viewing (7 out 11 features, loading ≥0.2). Conversely, PC1 in blank screen viewing loaded on the maximum length and variability of gaze steps, as well as on the number of flips on the Y axis, features that were mainly related to PC2 and PC3 during visual exploration (6 out of 7 features, loading ≥0.2). This was also confirmed quantitatively by running a linear regression model with PC1 during blank screen viewing as a dependent variable, and PC1−3 of image viewing (as well as their interactions) as predictors. This model showed that PC3 during image-viewing significantly predicted PC1 during blank screen viewing (*t* = 2.98, *p* = 0.004).

**Fig. 7:**
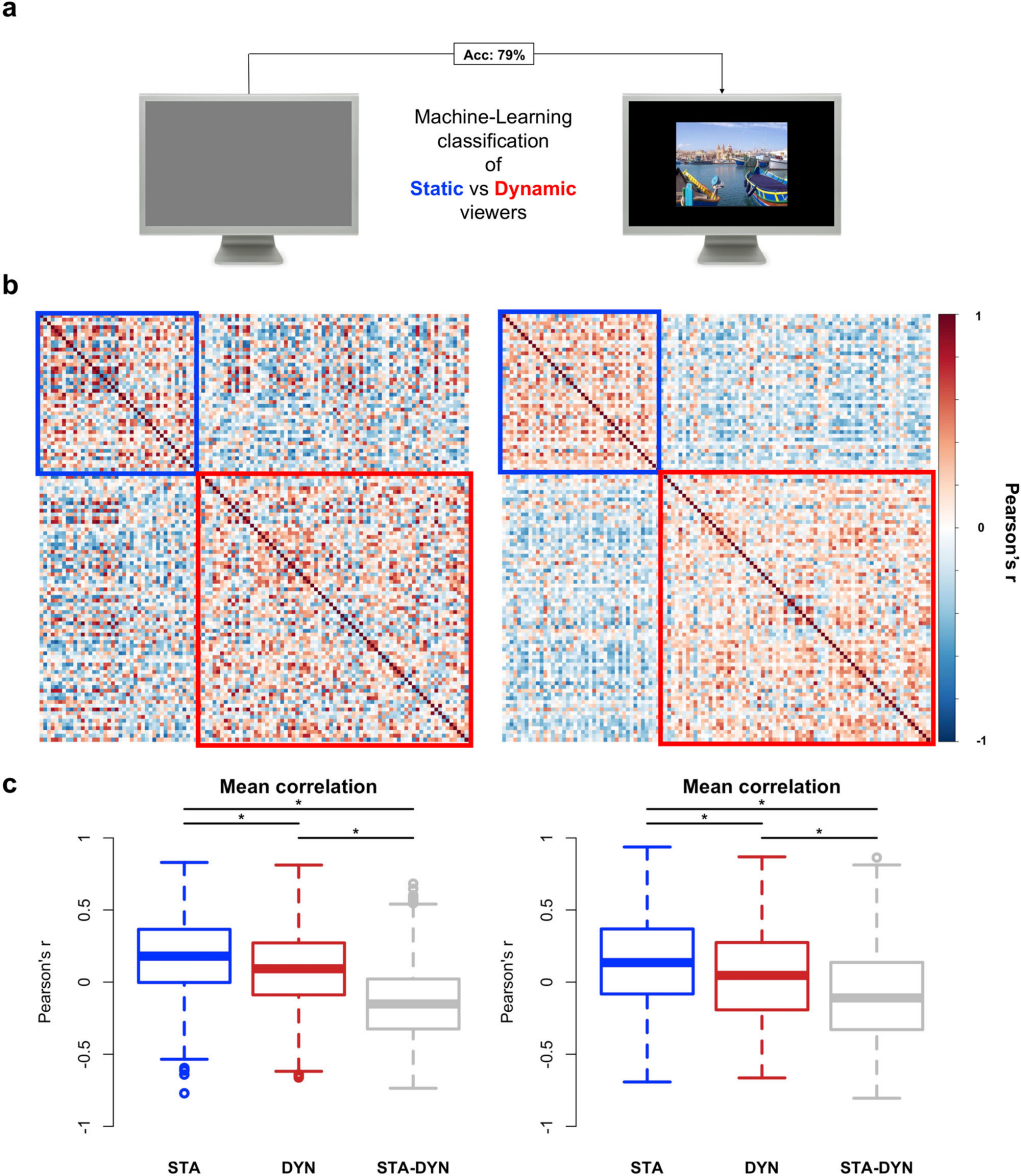
Subjects similarity in image-viewing and blank screen viewing. **a** Blank screen viewing eye movement features were extracted and used to predict individual subject labels (Static vs. Dynamic) by means of a random forest classifier. The algorithm was trained on features extracted from the blank screen viewing condition and tested on cluster labels extracted while participants were exploring visual scenes, in a cross-classification design. The model showed 79% accuracy in cluster classification from blank screen viewing features. **b** For each pair of subjects a Pearson’s *r* is computed between the vectors of *z*-scored features extracted from the image-viewing task (right) and the blank screen viewing condition (left). *X* and *Y* axes indicate subjects. The colour of each cell indicates the Pearson’s correlation value, while the coloured squares indicate the cluster (i.e., the visual exploration style; blue = static viewers; red = dynamic viewers). **c** Boxplots showing the comparison between the mean correlation within each group (static = STA, *n* = 42; dynamic = DYN, *n* = 72) and between groups (STA-DYN). STA = within-group correlation in static viewers; DYN = within-group correlation in Dynamic viewers; STA-DYN: between-groups correlation. * = Bonferroni-corrected significant difference tested by means of a two-sample t-test.

Next, we used blank screen viewing eye movement features to predict individual subject labels (Static vs. Dynamic viewers) using a multivariate Random Forest algorithm in a cross-classification design. That is, the algorithm was trained on features extracted in the blank screen viewing condition and tested on cluster labels extracted during the image-viewing task. The model showed an accuracy of 79% (*p* < .001; 95% C.I. [71.3–87.0]) in predicting cluster labels from features extracted from blank screen viewing (Fig. 7a; features importance is shown in Supplementary Fig. 11). We also checked the stability of viewing styles between image-viewing and blank screen viewing conditions and we found that the individual viewing style was maintained in 68% of cases (Supplementary Fig. 12). Inspection of the between-subjects correlation matrix of eye movement features during visual exploration and blank screen viewing shows that individuals tend to correlate significantly more with members of the same cluster (within) than with members of the other cluster (between; Fig. 7b, c; all Bonferroni-corrected t-test *p* < .001). Moreover, the mean correlation among subjects within the Static cluster was significantly stronger than among subjects within the Dynamic cluster, both in image-viewing (*t*[1451.1] = 8.76, *p* < .001) and blank screen viewing (*t*[1422.9] = 6.59, *p* < .001). Importantly, the structure of the between-subjects similarity in visual exploration (Fig. 7b, left matrix) significantly correlated with that in blank screen viewing (Fig. 7b, right matrix; Pearson’s *r* = 0.37, *p* < .001). These findings show that the visual exploration style found during free viewing of natural scenes is identifiable even in absence of visual stimuli.

### Eye movement dynamics correlate with cognition and personality

The final analysis investigated whether eye movement spatiotemporal features (as indexed by PC1 scores) were related to individual characteristics, namely demographic information (i.e., age, sex, and education), cognitive scores (i.e., inhibition, visuospatial and verbal memory), and personality traits (i.e., Big Five scores).

Indeed, an emerging body of research suggests a link between eye movements and personality traits^16,36–39^, with Openness related to longer fixations^37^.

The full regression model (see Cognitive-Personality model in Supplementary Table 5) included all test scores listed in Supplementary Table 1 as predictors, with the exception of depression and anxiety scores (DASS) and visuospatial constructional abilities scores (copy of the Rey−Osterrieth Complex Figure; ROCF). The DASS scores were not included in the model since they were employed only to exclude participants with high levels of anxiety, depression, and/or stress, to avoid biased eye movement data. The copy of the ROCF was excluded because it shows ceiling effect in healthy participants and for our purposes it was administered only to test the delayed recall. The model was significant (*F*[16,81] = 1.84, *p* = 0.03, adjusted-*R*^2^ = 0.12) with a significant effect of Age (*t* = 2.66, *p* = 0.009; Fig. 6c) and impulsivity (i.e., Stroop test score; *t* = −2.36, *p* = 0.021; Fig. 6d), and trend significance for the NEO-FFI subscale Openness (*t* = 1.93, *p* = 0.057). Specifically, dynamic viewers were younger (range of the whole sample: 19–34 years old), showed higher impulsivity (i.e., lower inhibition of automatic responses at Stroop test), and a non-significant tendency for being less open. The model was validated using the split-half procedure described above with 1,000 iterations (Fig. 6e).

## Discussion

In this study, we measured eye movements in a large sample of healthy participants during visual exploration of many real-world scenes. We found that eye movement spatiotemporal parameters were strongly correlated across pictures and participants, with three components explaining roughly 60% of the variance of eye movement dynamics. This low dimensional structure of eye movement patterns, especially the duration and number of fixation (PC1) identified two viewing styles: static and dynamic. The inter-individual variability of PC1 scores was significantly predicted by the similarity of gaze step length distribution to a power-law, an intrinsic property of dynamical systems, but not by the saliency or semantic content of the visual scenes. In addition, static and dynamic viewers could be identified by the pattern of eye movement features while participants looked at a blank screen, and they differed in their cognitive profile.

Herein, we discuss two main results: the low dimensionality of eye movement spatiotemporal features during visual exploration, and the role of intrinsic dynamics vis-a-vis sensory salience and semantic information in guiding visual exploration style.

The low dimensionality of eye movements is not an entirely novel result. Poynter and colleagues^9^, in a study on *n* = 40 subjects, found that eye movement parameters correlated across different laboratory tasks (e.g., sustained fixation, search, Stroop), and could be summarized with a single factor, putatively related to visual attention. Their factor loaded on the duration and frequency of fixations, which is also an important component of our PC1. Using a larger set of features, we separated two clusters of observers, static and dynamic, who differed not only in terms of the rate or duration of fixation, but also pupil diameter, spontaneous viewing time, amplitude and number of gaze steps, and number of gaze flips (Fig. 4). The assignment to one cluster or the other was stable (>90% accuracy) across different sets of images.

Static viewers showed less frequent but longer fixations, longer exploration time, larger and more numerous gaze steps, more gaze flips (i.e., change of gaze direction), smaller pupil diameter, as well as a distribution of gaze steps closer to a power law. Moreover, they spent less time on parts of the images that were rich in semantic and saliency information. Dynamic viewers showed the opposite pattern. Intuitively, static viewers better approximated a power-law distribution because they showed more small amplitude and relative few long-range gaze steps, while dynamic viewers made a more balanced combination of short and long gaze steps.

The covariance of fixation duration and gaze step distribution is consistent with an interdependent control process^40^. At the neural level, fixation and saccadic activity are inter-related at multiple levels in the brain (frontal eye field, superior colliculus, brainstem^41–43^). At the cortical level, different neural systems, the dorsal and ventral attention networks^44,45^, control focal processing vs. re-orienting to novel locations.

Visual processing occurs during fixations, hence a longer fixation time in static viewers may imply more-in-depth processing of fewer stimuli. Conversely, dynamic viewers may look more rapidly, and more superficially, to more items in a visual scene. This interpretation is also consistent with the observation that PC1 scores were related to impulsivity, i.e., the ability to inhibit an automatic response. Specifically, dynamic viewers tend to be more impulsive than static viewers. This is in line with previous literature linking impulsivity to voluntary control of fixation behaviour^46^.

The presence of low dimensionality and individual styles in human cognition that defines inter-individual variability is consistent with other recent findings. For instance, a recent study classified individuals along the Big-Five dimensions of personality based on patterns of eye movements in real life (walking on campus)^16^. Similarly, studies of human mobility have revealed two distinct styles^47^ during walking from one location to another in a city: “Returners” who tend to walk back-and-forth nearly always taking the same trajectory, and “Explorers” who explore more frequently new locations in their route. The authors showed also a social bias in the mobility profile, with a tendency to engage more socially individuals with a similar mobility profile.

In the field of reward, we have recently shown that the temporal discount functions in a large group of healthy subjects (*n* = 1200) show a Pareto optimality distribution that defines three archetypes: people who always wait for larger rewards; people who always take immediately; and people who take immediately when the reward is large^48^. The existence of different styles may reflect trade-offs in cognitive or physical traits that have been selected during evolution to maximize specialized performance, similarly to what is shown in other fields such as animal behaviour^49^ or biological circuits^50^.

Next, we asked: what controls the low dimensionality of eye movement patterns across subjects? We first quantified sensory salience using a classic saliency model^1^, while semantic information was quantified based on a deep learning neural network^28^. The amount of saliency and semantic information within fixated locations were then used as predictors of PC1 scores, along with a measure of visual scanning topography (Shannon entropy of eye movements), and the distance of each individual eye movement distribution to a power law (KSD; see “Methods” section). The presence of power-law dynamics in behaviour (including eye movements), as well as in neural systems^24^, is thought to reflect intrinsic dynamics^34,35^. Surprisingly, we found that saliency or semantic information did not predict significantly PC1 scores (nor PC2). It is important to note that this result is not due to averaging of saliency or semantic information across pictures, thus leaving only shared information. Rather, estimates of saliency and semantic information were computed fixation by fixation (i.e., amount of saliency/semantic information collected by the observer in each fixation), therefore taking into account eye movement patterns in each picture separately.

On the other hand, saliency and semantic maps significantly predicted the topography of fixations, as expected from previous literature. Taken together these results support and extend previous literature suggesting that saliency models accurately predict eye movements behaviour during free-viewing in terms of fixation topography^51–53^. This is in line with the assumption that when an observer is not engaged in a specific task eye movements will be directed to regions of higher saliency^51,52^.

However, saliency plays a lesser role in predicting the spatiotemporal properties of eye movements, i.e., their dynamics (as previously shown for scanpaths^51^). Here, several results pointed to the importance of endogenous factors. First, the components mainly describing spatiotemporal eye movements features that are independent of image content (e.g., image category). Secondly, the low influence of saliency and semantics in explaining PC1 variability. Thirdly, the similarity of the spatiotemporal latent variables during free viewing of static natural images and blank screen. Importantly, we find that the distance of the distribution of gaze shifts from a power law (KSD) predicts about 20% of the PC1 score variability across subjects. In addition, KSD also contributes albeit less strongly than saliency and semantic information to the topography of fixations.

Power laws are ubiquitous in the world, as well as in the brain where they are thought to reflect neurobiological constraints imposed by anatomical connectivity and neural dynamics. Power laws have been described in fMRI, EEG/MEG, local field potentials, and single-unit activity^54–56^. Moreover, behavioural performance fluctuations also follow a power law, including eye movements^40^, and tend to correlate with slow and fast neuronal activity. Interestingly, the power-law exponents of behaviour and neural activity are correlated across individuals both during task and rest^57^. Therefore, we posited that a similar link may occur between eye movement dynamics and neural dynamics, even spontaneously at rest (i.e., during blank screen viewing). This implies that resting dynamics have an influence on how we move the eyes during visual exploration, thus potentially revealing stable, biologically determined, traits of the observer^58^.

This was confirmed in our recordings of eye movements to a blank screen. We found, in this case, three components that explained a similar amount of variance (~50%) with the most variance explained by gaze step amplitude (gaze step length PC1: 29% variance), and the least variance explained by fixation duration and frequency (PC3: 9% variance). Hence, the features defining the three components resembled those found during visual exploration, but their relative weight differed. During exploration, eye movement variability was mainly explained by fixation duration; during blank screen viewing, the variability of eye movement spatiotemporal features was mainly explained by the amplitude of gaze steps. This indicates that similar components are active in both situations, but that visual exploration gently moves the attractor space of eye movement parameters. This finding is in line with the similarity of brain activity topography at rest and during tasks^59,60^, with the relative correlation within and between networks adjusted during different tasks^60–62^. This is consistent with the idea that spontaneous neural dynamics function as a spatiotemporal prior constraining the parameters space of task-evoked activity^63,64^.

Our results are consistent with a previous small-scale study (*n* = 15) in which visual exploration eye movements were compared to eye movements recorded in darkness^10^. However, eye movements in darkness could reflect several factors not directly related to spontaneous visual exploration dynamics, such as posture-related information^65^ or memory-related processing^66^. Also, pupillary responses are not controlled in the darkness. Other small-scale studies used a similar blank screen condition during a memory retrieval task^67^ or while hearing sentences about a previously presented scene^68^. To the best of our knowledge, our work represents the first large-scale study in which spontaneous eye movement dynamics are compared to those recorded during exploration of many real-world visual scenes, and the first to show that characteristics of eye movements at rest (i.e., during blank screen viewing) can be used to classify different styles of visual exploration.

Regarding the present study’s limitations, the sampling rate of the eye tracker (i.e., 120 Hz) did not allow us to investigate in detail the dynamics of microsaccades that are an important mechanism of fixation. Visual exploration could be also studied in more natural conditions without the use of a chin-rest support using algorithms for head movements correction, or wearable eye-trackers. The blank screen viewing period of observation was short (30 s prior to the presentation of the first image) so that we cannot rule out that some degree of expectation did influence the results. Also, longer blank screen viewing periods would allow the detection of slower fluctuations of eye movement patterns, as well as pupillary responses that are related to vigilance fluctuations and could significantly impact intrinsic activity^69^.

In conclusion, eye movement features during free visual exploration are correlated across subjects, and cluster people in two phenotypes depending on their style of exploration. The degree to which the distribution of gaze steps length resembled a power-law was the strongest predictor of the visual exploration style. We speculate that this could suggest the existence of neurological constraints that drive visual exploration behaviour and predict individual differences, e.g., patterns of anatomical connectivity and/or neural dynamics.

Another related implication of this work would be its potential application in clinical populations. For instance, some authors have shown that neurodegenerative disorders are associated with specific patterns of eye-movements features^70^, but these studies have mainly used laboratory tasks (e.g., anti-saccades tasks), with some investigations during reading^71,72^, and not focused on intrinsic dynamics. It is possible that alterations of eye movement intrinsic patterns may represent an early biomarker of neurodegeneration.

## Methods

### Subjects

A sample of 120 students was recruited at the University of Padova (mean age = 23.4, SD = 2.42; 49 M). All participants had normal or corrected-to-normal (i.e., glasses, *n* = 54) vision. We excluded individuals with excessive data loss, defined as less than 50% of usable data in more than 25% of trials (*n* = 3 individuals excluded). Moreover, two further participants were excluded due to the interruption of the experimental session for a panic attack in one case, and for eyes irritation in the other case. Finally, one participant was excluded because of colour-blindness revealed after the experimental session was completed. Thus, 114 out of 120 participants were included in the final sample (mean age = 23.52, SD = 2.45, 67 F). All participants signed an informed consent before the experimental session and after it, they received a remuneration of 10€ for their participation. The study was approved by the Ethical Committee of the University of Padova.

### Experimental design

Each participant took part in a single session composed of five phases (total duration: 2 h). In the first phase, participants were asked to look at a grey screen without any stimulation for 30 s. Participants were just told to freely move their eyes within the screen boundaries.

In the second phase a set of 185 images of scenes selected from the Places 365 database (see the *Stimuli* paragraph for details about the dataset and the stimuli selection). Stimuli subtended 27.1° × 20.5° (width × height) degrees of visual angle on a screen subtending 31.6° × 23.9°. Participants were instructed to freely look at the pictures in a self-paced design (for min 2,000 ms−max 10,000 ms; 1500 ms ITI) and to move to the next trial by pressing the spacebar. Moreover, they were informed that they would be asked some questions at the end of the task. After the first half of the images was presented, participants had a 10 min break to let them relax and rest their eyes. Once all the pictures were presented, participants had another 5 min break before the third phase in which they were asked to recall the five repeated images. Participants were requested to describe each image for 3 min as accurately as possible while their verbal description was recorded by means of a voice recorder. During the recall, participants were presented with the same grey screen adopted in phase 1. For the purpose of the present paper, only phases 1 and 2 have been considered.

### Stimuli

The stimuli used in the present experiment were real-world scenes selected from the Places dataset^28^, a scenes dataset designed to train artificial systems for image recognition. Specifically, the dataset we used in this experiment is the validation set of the Places365-Standard dataset (the dataset can be downloaded here: http://places2.csail.mit.edu/download.html). All images in the dataset were categorized according to three hierarchical levels. Level 1 was the most general and subdivided the images into three categories: indoor, outdoor man-made, and outdoor natural. In Level 2, each of the categories in Level 1 was split into four to six subcategories (e.g., for Level 1 category “indoor”, Level 2 subcategories examples are “shopping and dining” and “home or hotel”). Finally, Level 3 encoded 365 specific categories describing the type of scene (e.g., art gallery, bakery shop, etc.)

For the purposes of the present work, only Level 1 categorization was chosen, moreover, images were coded through an additional dimension, that is whether they depicted human beings or not. Thus, six categories were finally considered (i.e., indoor manmade with humans, indoor manmade without humans, outdoor manmade with humans, outdoor manmade without humans, outdoor natural with humans, outdoor natural without humans) and 30 images for each category were chosen (e.g., outdoor manmade with humans; Supplementary Fig. 2). The final set of images was composed of 180 items with the add of five further images for the recall phase purpose. These images were taken from all the above-described categories but outdoor natural images without humans as this type of images showed a very low number of recallable details. Details about the image selection process are reported in Supplementary Fig. 1.

### Assessment of behaviour and personality

Participants were tested after the eye-tracker data acquisition was completed. For the cognitive assessment, we decided to focus on memory (visuospatial long-term memory, working memory) and executive functions (inhibition/impulsivity) as these domains seem to mainly influence visual behaviour^73^.

The cognitive tests employed to assess the described domains were the Digit Span (forward and backward)^74^, the brief version of the Stroop Test^75^, and the ROCF^76^. Moreover, we asked participants to fill a form sent by e-mail which included three questionnaires. One of these was a personality questionnaire based on the Five Factor Model^77^, the Neo Five Factors Inventory (NEO-FFI)^78^ which evaluates the following factors: Extraversion, Agreeableness, Conscientiousness, Neuroticism, and Openness to Experience. A number of studies have shown a link between personality factors and several aspects of eye-movement such as the pattern of fixations^79^, the number of fixations, their duration, and dwelling time^37^. Starting from this point, in a recent paper^16^ authors demonstrated that personality traits can be predicted from a set of visual features by means of a multivariate machine-learning approach. This result suggests an important role of individual characteristics on visual behaviour. Furthermore, in the present study, we assessed impulsivity in complex behaviours by means of the behavioural approach system and behavioural inhibition scale (BIS-BAS)^80^. The relation between impulsivity and eye-movements has been previously pointed out in literature^37^. The information extracted from this questionnaire can be seen as complementary to those taken from the Stroop Test, thus, taken together, they allow to investigate impulsivity both from cognitive and behavioural points of view. Finally, the 21-items version of the Depression Anxiety Stress Scale (DASS-21)^81^ was used to control for participants’ state anxiety, as it can have influence visual behaviour^82^. None of the participants was discarded for excessive state anxiety score. Moreover, since some participants were students of psychology, we checked their knowledge of the administered tests using a three-point scale (0 = No knowledge; 1 = Theoretical knowledge; 2 = Theoretical and Practical knowledge). No effects of previous knowledge emerged on the subsequent models.

### Eye-tracker data acquisition, pre-processing, and features extraction

The eye-tracker adopted was the Tobii T120 (Tobii Technologies, Danderyd, Sweden) which allows to acquire gaze data with a 120 Hz sampling-rate (or every 8.3 ms). Participants were seated at a fixed distance of 60 cm from the screen, and their head-movements were limited by a chin-rest.

Raw eye-tracking data were minimally pre-processed. We included in the analysis only gaze samples in which both eyes were assigned the highest validity value (i.e, validity code of 0, indicating that the eye is found and that the tracking quality is good). Then, we extracted a large set of features encoding various characteristics of eye-movements to describe visual behaviour in an exhaustive way, as done in other recent studies^16^.

For each participant, a set of 58 features was extracted (Supplementary Table 2) which encoded four main sources of information: fixations, pupil diameter, gaze steps, and exploration time. Statistics over fixations (e.g., mean duration) are frequently employed in eye-tracking studies^37^. In the present study, fixations were detected using a velocity-based threshold algorithm^83^ (detection threshold lambda = 15), which is considered adequate and robust across several testing conditions^84^. From a cognitive point of view, fixations represent information processing and their duration is correlated with the depth of cognitive processing^85^. Pupil diameter (e.g., mean pupil diameter of the left eye) was considered since it is not only related to environmental light and vigilance, but also to a variety of cognitive processes such as attention^86^ and cognitive load^87^. Gaze steps statistics (e.g., mean gaze step length, number of flips on *x* and *y* axes) were computed after extracting gaze steps from raw gaze data as the Euclidean pixel distance between two consecutive gaze positions^18^. Notably, the use of this metric allows to avoid the distinction between saccades and microsaccades, as both types of eye movements are thought to be controlled by the same neuronal mechanisms^43^. Finally, statistics describing exploration time were taken into account (e.g., mean duration of pictures’ exploration). Moreover, for fixations and gaze steps, additional features were extracted which encoded their temporal course (e.g., mean fixation duration in the first, second, third, and fourth quarter of exploration time). This set of features is largely overlapped with those adopted in previous studies that examined the relationship between eye movements and individual characteristics^16^. The choice of these features was made to capture the spatiotemporal features of eye movements and fixations rather than their spatial distribution.

### Eye-movements data reduction

A principal components analysis (PCA) was performed on the features matrix (114 subjects × 58 features) to reduce it to fewer meaningful components. Oblique rotation (Promax) was adopted because of the correlation between the features. To select the optimal number of components we adopted the Kaiser’s criterion^88^ and selected only components with eigenvalues higher than 1. In addition, to be selected a component had to account for a percentage of the variance of at least 10%.

For the image-viewing task, according to the selection criteria and after visual inspection of a scree plot, a three-component solution was chosen. The first three components globally explained roughly 60% of the variance. The first component (explained variance: 31.1%) mainly loaded on fixations duration, the second component (explained variance: 16.5%) mainly loaded on exploration time, number of steps, and number of flips (i.e., changes of direction on *X* or *Y* axis), finally, third component (explained variance: 12.2%) mainly loaded on steps’ length.

For the blank screen viewing phase, a separate PCA analysis was done after computing the same set of features as before. The reason of this is that in the blank screen viewing condition the exploration time was basically the same for all participants. Since fourteen participants showed missing data in some fixation-based features (e.g., due to a single central fixation), only 100 participants were included in this analysis. Moreover, exploration time-based features were removed, as blank screen viewing had the same duration (30 s) for all subjects. Thus, the PCA on blank screen viewing data was performed on 100 subjects and 53 features.

Moreover, in the PCA on the blank screen viewing features, we decided to include the first three components regardless of the amount of explained variance, to match the structure of the previous PCA on the image-viewing task. The first component (explained variance: 23.4%) mainly loaded on the number of steps, number of flips, and steps’ length variability. The second component (explained variance: 19%) mainly loaded on pupil diameter and steps’ length, while the third component (explained variance: 8.4%) mainly loaded on fixations duration (Supplementary Fig. 10).

Interestingly, the most important features in the blank screen viewing condition were mainly included in the third component extracted from the image-viewing task. This suggests that the importance of fixation-related features was lower if compared to the image-viewing condition, while more importance was assigned to pupil diameter and steps’ length.

### Detection of clusters in visual behaviour and their interpretation

Preliminarily, the Silhouette method^89^ was applied to identify the optimal number of clusters in a data-driven manner, and suggested the existence of two clusters in our data. Then, a *k*-means cluster analysis with a *k* value of 2 was carried out. The reliability of the two clusters solution was tested by comparing different clustering solutions obtained from *k*-means and hierarchical clustering algorithms, using several distance metrics. The similarity between the clustering solutions was quantified by means of the Jaccard index (Supplementary Fig. 3) and revealed that the two clusters solution was the most reliable across different methods. Figure 3 shows the participants scores in the three-dimensional space defined by the first three principal components, coloured according to the cluster participants belonged to. The PC1 scores accounted well for the differences between the two clusters which were represented by a continuum. Subsequently, we wanted to investigate whether the different visual exploration styles were associated with differences in the topography of the visual exploration pattern (i.e., entropy), in the distribution of gaze steps (i.e., more power-law-like), and in the informational content of fixations (i.e., whether subjects paid more attention to saliency or semantic information).

First, for each participant, 185 heatmaps were created (i.e., one for each presented picture) representing the empirical gaze maps encoding the normalized number of times the gaze was centred in each pixel. The Shannon entropy was calculated for each heatmap.

Second, the distance (i.e., Euclidean distance) covered in each gaze step (i.e., gaze step length) was calculated and the distribution of their length was computed. Then, the subject-specific gaze step length distribution was fitted to a power-law distribution and their similarity was quantified by means of the Kolmogorov−Smirnov test, a well-known nonparametric test that is used to compare distributions^90^. Specifically, in our case, this test was used to investigate whether an empirical probability distribution (i.e., the subject-based distribution of gaze steps length) disagreed from a reference distribution (i.e., the power-law distribution), by quantifying the distance between these two distributions (KSD). The lower the KSD, the higher the similarity between the empirical distribution and the reference power-law distribution. Importantly, this procedure was applied to each individual gaze steps distribution independently, leading to a different power-law exponent for each participant.

Third, we wanted to quantify the influence of saliency and semantic information in shaping general properties of visual exploration of real-world scenes. To this end, we created two types of heatmaps for each image: (1) a saliency map created using the classical saliency model by Itti and colleagues^1^ implemented in the graph-based visual saliency (GBVS) Matlab toolbox^91^; (2) a semantic map created by means of a recently published algorithm based on a convolutional neural network (CNN: residual network)^28^. These maps were used to quantify, fixation by fixation, the quantity of saliency and semantic information included. We, therefore, calculated the mean amount of saliency and semantic information fixated by each subject. Supplementary Fig. 6 shows a graphical explanation of this procedure. All computed heatmaps were spatially smoothed using a 2° full width at half maximum (FWHM) Gaussian Kernel.

Three nested linear regression models were built with PC1 scores (obtained in the image-viewing task) as a dependent variable, and the measures described above as predictors. Specifically, the first model (M1) included only saliency in fixations, then in M2 we added semantic information, in M3 also Shannon Entropy of visual exploration was included, and finally, M4 included all previously mentioned predictors as well as KSD. The models were compared by means of a likelihood ratio test (LRT) to highlight the most plausible model given the data. The resulting best model (M4) was then tested on the whole sample, and its reliability and generalizability were tested by randomly splitting the sample into two halves, fitting the model on one half (i.e., the training set), and testing its prediction (i.e., PC1 score) on the other half data (i.e., the test set). This procedure was repeated 1,000 times and each time the correlation between actual and predicted PC1 values was collected (Fig. 6B).

Furthermore, we built a further linear regression model with the aim to investigate whether visual exploration styles (PC1 scores) were predicted by demographic information (i.e., age, sex, and education), cognitive (i.e., inhibition, visuospatial, and verbal memory) or personality traits (i.e., Big Five scores). The full regression model (i.e., including all predictors; Supplementary Table 5) was tested and validated by applying the same split-half validation procedure used before (with 1,000 iterations; Fig. 6E).

### Prediction of fixation topography from saliency, semantic information, and KSD

In this analysis, we aimed to test whether KSD could predict the topography of fixations. To this end, we first computed a FDM for each image and for each subject (resulting in 185×114 FDMs). A Gaussian kernel (FWHM = 2°) was then applied to approximate the width of the visual field saw by the fovea and to match the other maps. Then, we computed spatial KSD maps as follows: (1) since KSD is computed on a distribution of gaze-steps, it could not be computed at the pixel-level, thus we subsampled the image space (512 × 683 pixels) to a smaller (10 × 10) matrix; (2) from raw gaze data we extracted the vector of gaze-steps amplitude within each cell while watching an image at the subject-level, and we calculated corresponding KSD values, resulting in a 10 × 10 KSD matrix. This procedure was applied to all images and all subjects resulting in 114 × 185 KSD maps. Then, FDMs, saliency, and semantic maps were subsampled to match the size of the KSD maps. Finally, the resulting set of matrices were vectorized, concatenated, and used to build three mixed-effect models (MM1, MM2, and MM3) to quantify the contribution of saliency, semantic information, and KSD in describing the spatial distribution of fixations (FDMs). A graphical representation of this method is shown in Supplementary Fig. 9.

Each model included a random intercept for both images and subjects, and nested sets of fixed effects: MM1 included only saliency maps; in MM2 we added semantic maps, and in MM3 also KSD maps were included. The models were compared through LRT as described in the previous paragraph and the results of the best model (MM3) were discussed. Notably, the model comparison was run also within each subject independently, by building the same models with random intercept only for images.

### Machine-learning classification analysis of cluster labels from blank screen viewing eye-movements’ features

We investigated whether the features extracted during blank screen viewing were informative about the visual exploration styles that emerged while watching real-world scenes. To do so, we trained a Random Forest classifier to predict the two cluster labels (Static vs Dynamic, as determined in the image-viewing condition) from the blank screen viewing multivariate pattern of eye-movement features. We used a 10-fold cross-validation design, i.e., data were split into 10 folds, nine of which were used as training set, and one was left out and used as test set. This procedure was repeated for 10 iterations until each fold was used once as test set, resulting in a mean accuracy value indicating the proportion of participants correctly labelled.

Moreover, we computed a features correlation matrix between subjects, thus testing the interindividual similarity in the pattern of eye-movement’s features (Fig. 7B). As shown in the figure, the correlation is higher for participants falling within the same cluster (i.e., Static viewers or Dynamic viewers) than between participants with different visual exploration styles. Then, to test the reliability of this pattern of between-subjects similarity between blank screen viewing and image-viewing conditions, the Pearson’s correlation between the two matrices was computed.

### Statistics and reproducibility

Principal component analysis (PCA): performed on scaled and mean-centred full set of features extracted from the gaze data acquired during the exploration of images (*n* = 114), and during blank screen viewing (*n* = 100). Control analyses (PCA on image-viewing data): (I) splitting the images in odd/even, running a PCA on each subset and correlating the obtained PC1 scores with those found on the whole images set; (II) reconstructing the original features matrix (as well as individual patterns of features) from PC1, PC2, and PC3 scores and testing the accuracy of the reconstruction; (III) computing PC1 scores from features extracted from different image-categories (i.e., indoor, outdoor natural, outdoor manmade, with/without humans) and applying PC1 loadings calculated on all images to make scores comparable.﻿

K-means cluster analysis: on the same input of the PCA, after computing the optimal number of clusters (*k*) given the data, using the silhouette method. Control analysis: the chosen *k* was validated by comparing different clustering solutions (i.e., different *k* values) and different distance measures (e.g., Euclidean, Manhattan, etc.) and testing the similarity of different solutions, with the hypothesis that if applying different distance measures would give similar results (i.e., individuals clustered together), it would suggest a more reliable solution.

Linear regression models (visual exploration features): a set of models was built to explain PC1 scores. Specifically, four nested models were compared by means of a LRT which allows to determine whether adding a predictor to a simpler model would significantly improve model fit. Control analyses on the best model resulting from the LRT: (I) split-half reliability design in which 57/114 participants were randomly selected to fit model parameters and the other half was used as test set. This was repeated for 1,000 iterations and for each iteration the Pearson’s *r* coefficient between actual and predicted PC1 scores was collected; (II) the reliability of the main effect of the model (i.e., KSD) was checked by recomputing KSD using different gaze-step thresholds (0.2°−8.1°), that is gaze-steps smaller than each threshold were iteratively removed from the computation.

Linear regression model (demographic, cognitive, and personality information): only the full regression model including all test scores and demographic information was built. Control analysis: the model was validated using the split-half procedure described above with 1,000 iterations.

Linear mixed-effects models: we built a set of mixed-effects models to predict fixations topography (i.e., FDMs) from KSD, saliency, and semantic maps. Three nested models were compared by means of a LRT and pointed out that adding KSD to a model including saliency and semantic information improved model explanatory power of the topographical distribution of fixations. Each model included a random term (i.e., random intercept) for both individuals and images. Control analysis: in order to assess reproducibility, the effects of interest were also measured at the individual level (i.e., within each subject separately). The same models were built with the only difference that the random intercept was set only for images.

Machine learning classification: we classified subjects as Static vs. Dynamic viewers from features computed while viewing a blank screen by means of a multivariate Random Forest classifier. Control analyses: (I) we checked the stability of viewing styles between image-viewing and blank screen viewing conditions by computing PC1 values obtained in the two conditions (applying loadings obtained in the image-viewing condition to make scores comparable); (II) we compared the between-subjects correlation of eye movement features (during visual exploration and blank screen viewing) with members of the same cluster vs. with members of the other cluster.

Finally, to foster reproducibility, in all seed-based analyses (e.g., PCA) we used seed = 1234.

### Reporting summary

Further information on research design is available in the Nature Research Reporting Summary linked to this article.

## Supplementary information

Supplementary Information

Reporting Summary

## Data Availability

The data that support the findings of this study are available from the corresponding author on reasonable request. The source data used for the main figures are available at https://osf.io/2rkx9/.

## Code availability

All codes used for data analysis are available from the corresponding author on reasonable request.
