# Supplementary material for: Visual exploration dynamics are low-dimensional and driven by intrinsic factors
Source: Commun Biol. 2021 Sep 17;4:1100. doi: 10.1038/s42003-021-02608-x (PMC8448835; doi:10.1038/s42003-021-02608-x)
Supplement: Supplementary file 1 — Supplementary Information [file 42003_2021_2608_MOESM1_ESM.pdf]

Supplementary Information

Visual exploration dynamics are low-dimensional and driven by intrinsic factors

Andrea Zangrossi, Giorgia Cona, Miriam Celli, Marco Zorzi, and Maurizio Corbetta

Supplementary Figures

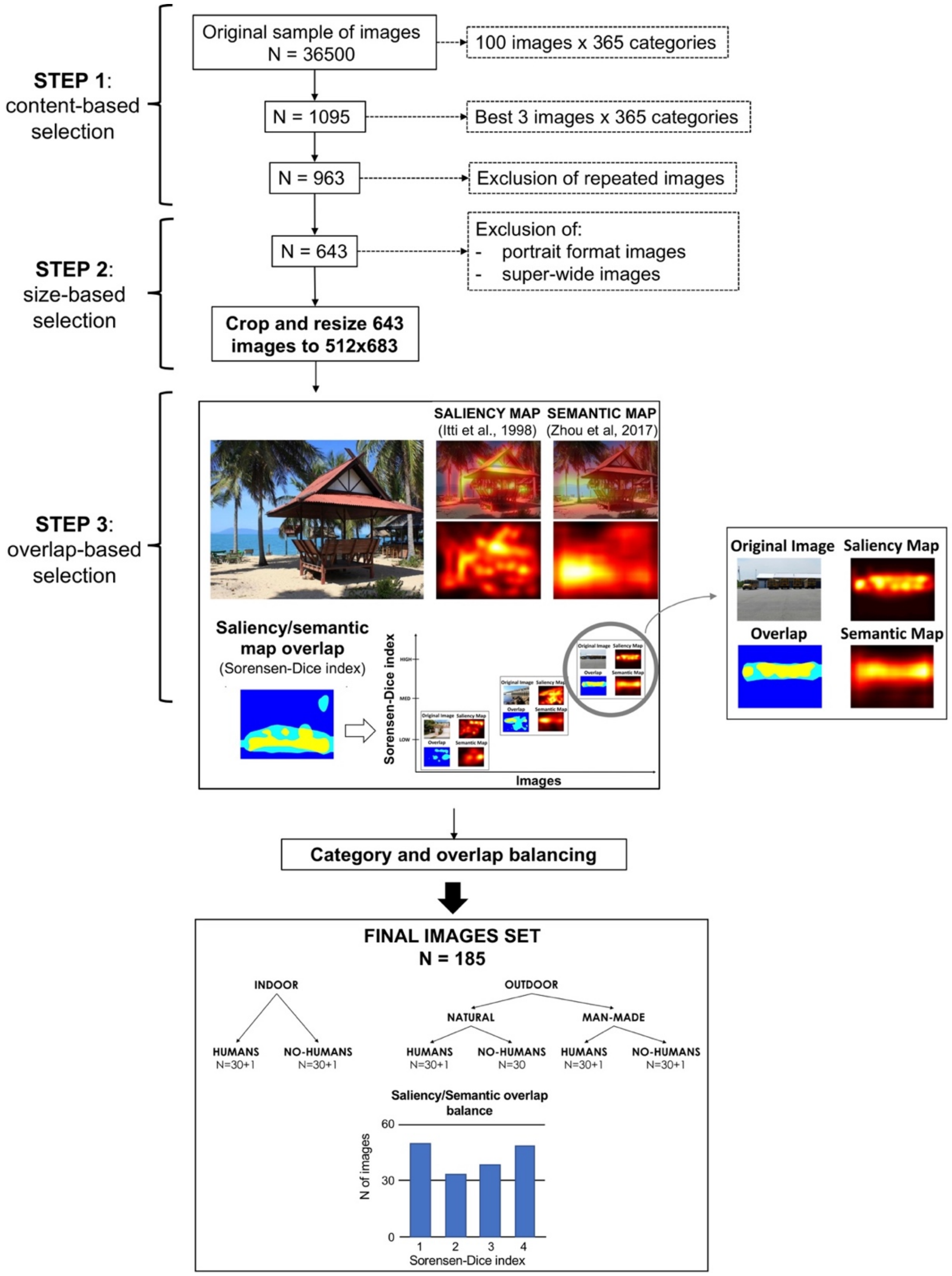

Supplementary Figure 1. Images' selection flowchart. The pipeline describes the procedure used to select the final sample of 185 images from the original set of 36500.

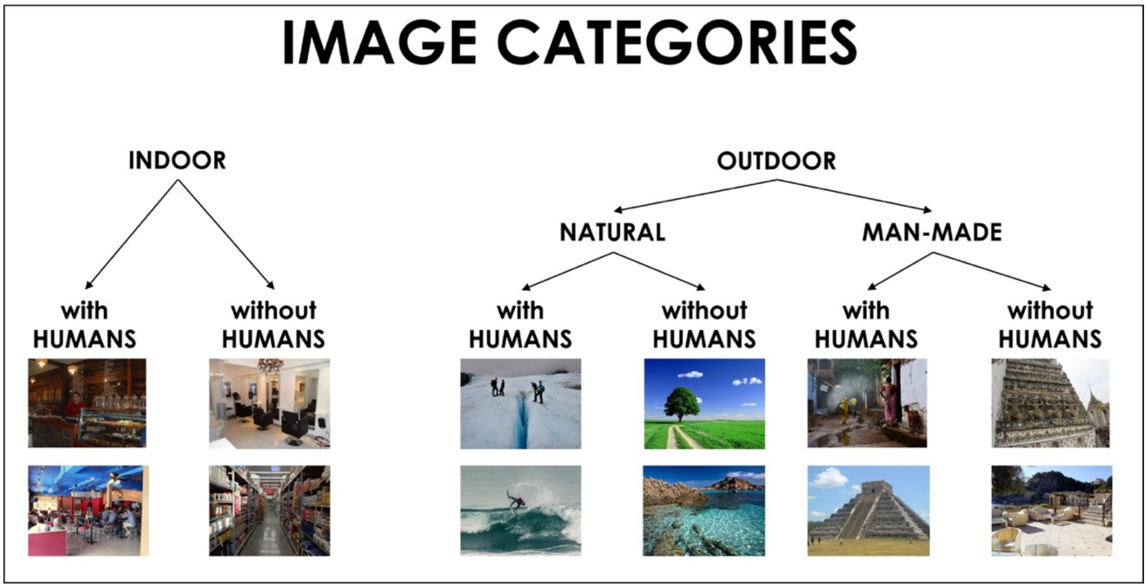

Supplementary Figure 2. Examples of images from each category. Each category was composed of 30 images (total: 180 images). Five more images were extracted to test the recall memory (not discussed in this paper).

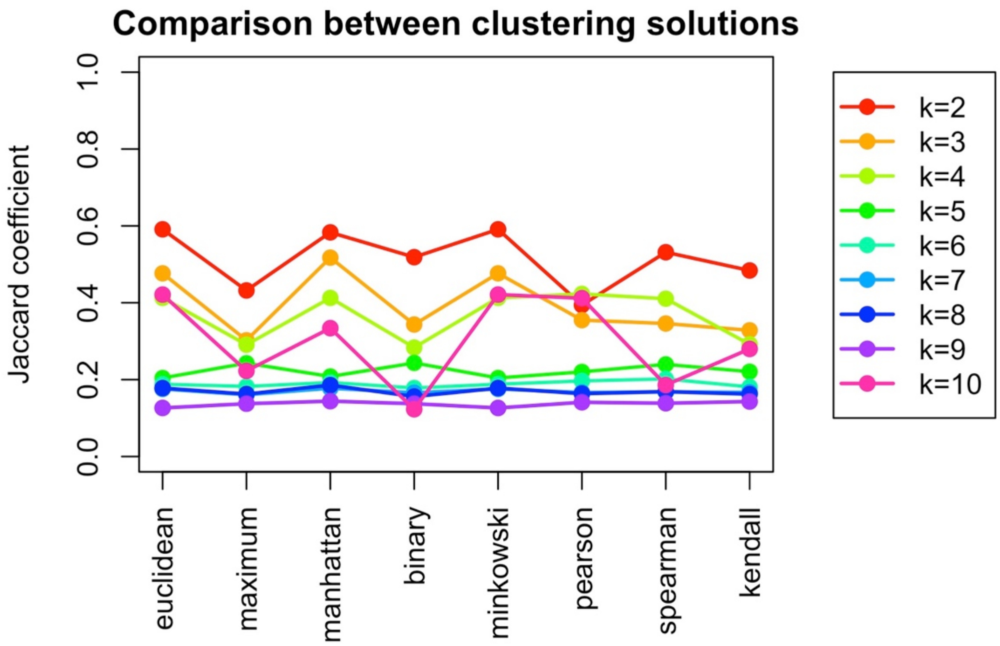

Supplementary Figure 3. Comparison between clustering solutions. Comparison between the clustering solution obtained using k-means and hierarchical clustering with different distance measures and different k values. The similarity is quantified by means of the Jaccard coefficient.

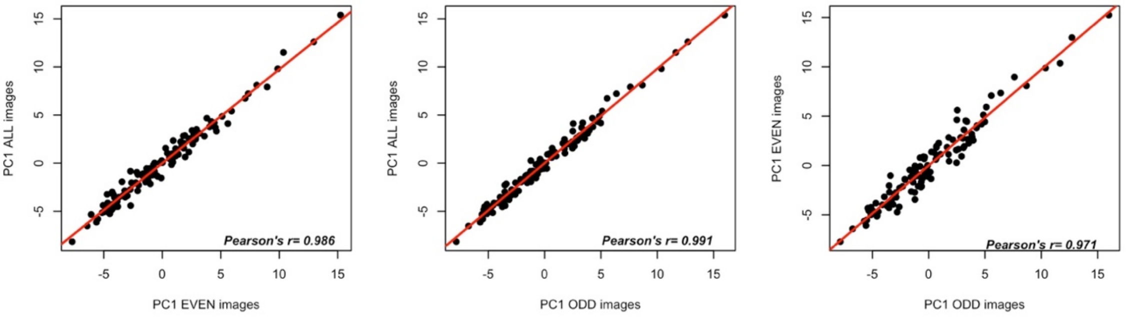

Supplementary Figure 4. Reliability of PC1 scores in different set of images. Correlation between PC1 individual scores (N=114) obtained on even and odd images (right), even and all images (left) and odd and all images (center).

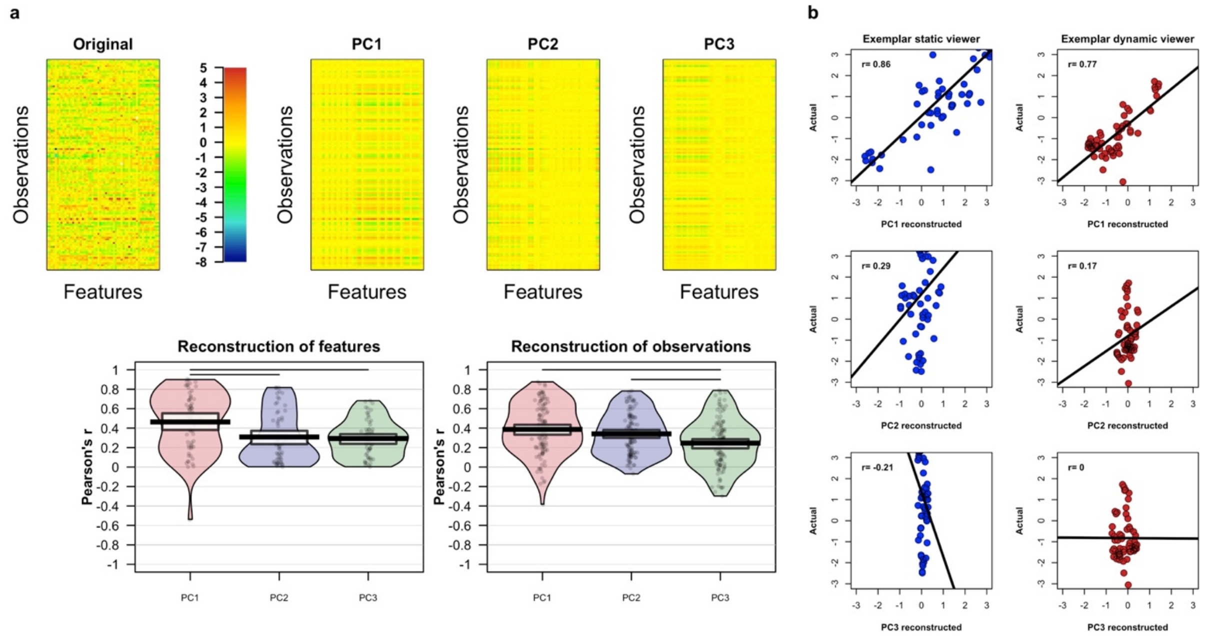

Supplementary Figure 5. PCA-based reconstruction of features. a. The original z-scored features matrix (top left) was reconstructed from PC1, PC2 and PC3, respectively, and the similarity between the original matrix and the reconstructed ones was evaluated separately for features (columns) and observations (rows) by means of Pearson's r. The violin plot shows the comparison between the distribution of Pearson's r correlation coefficients between original features/observation vectors and their reconstructions made from PC1, PC2 and PC3. The reconstruction of features based on PC1 was significantly more similar to the original features' matrix compared to both PC2- and PC3-based reconstructions. The reconstruction of observations based on PC1 was significantly better to those based onPC3, while the difference with PC2 was not significant. **b.** Reconstruction of the pattern of features of two exemplar observers (one Static and one Dynamic) from PC1 (top row), PC2 (center row) and PC3 (bottom row). The PC1-based reconstruction showed the highest similarity (Pearson's r) with the original pattern (r=.86 for the Static viewer and r=.77 for the Dynamic viewer).

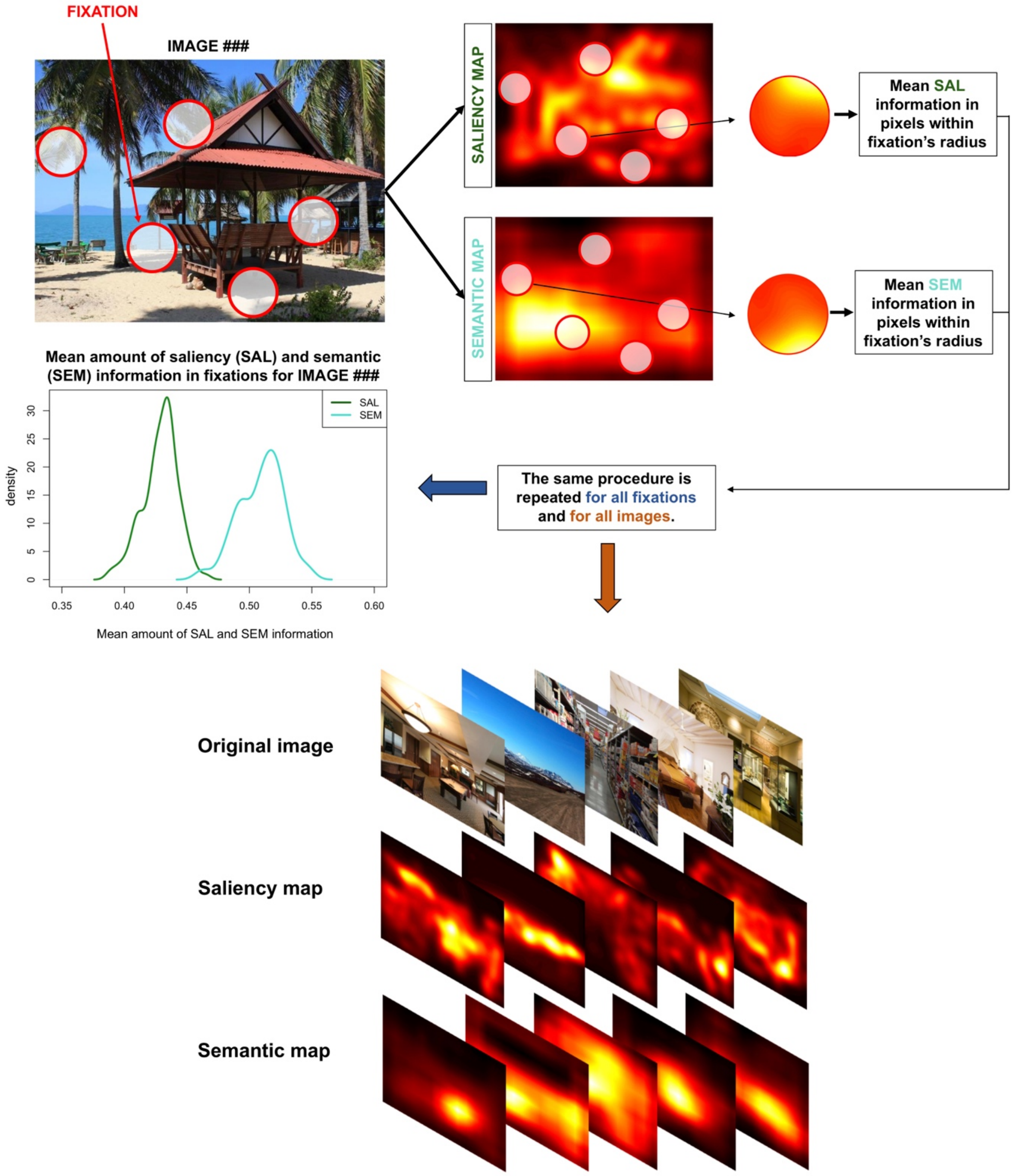

Supplementary Figure 6. Computation of saliency (SAL) and semantic (SEM) information within fixations. The figure represents the procedure we followed for the computation of SAL and SEM metrics, which were included in the regression models. For each fixation the saliency and semantic map values of pixels included in the fixation radius were averaged. Then, for each participant the average of all mean SAL and SEM values in his/her fixations was computer. Thus, in the regression model a single value of SAL and another of SEM was obtained for each participant. The lower part of the figure shows examples of saliency and semantic maps computed from five images.

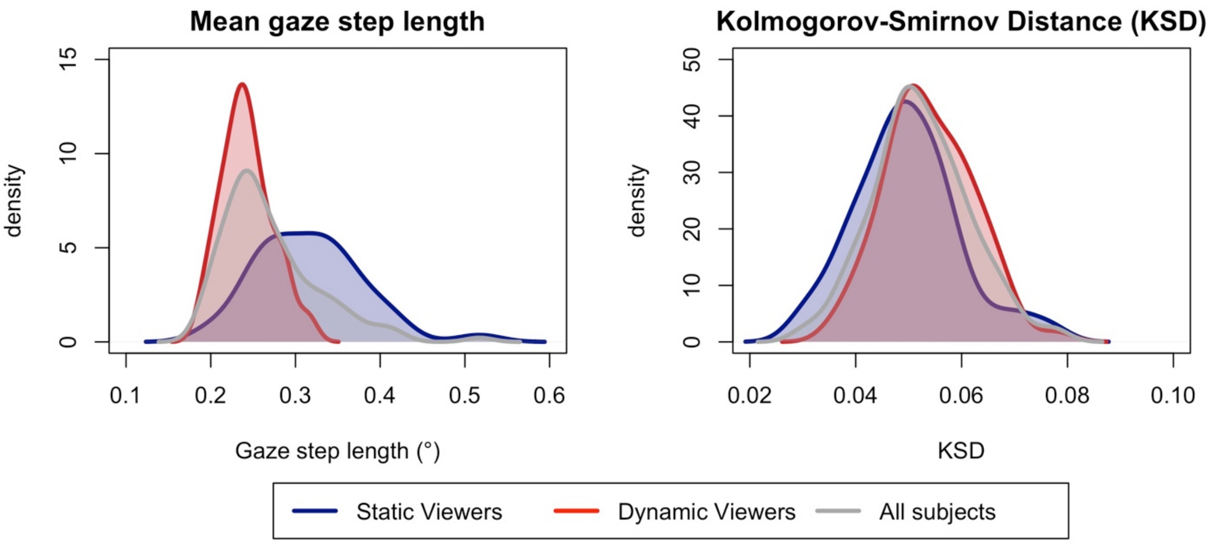

Supplementary Figure 7. Gaze step distribution (length) and similarity to power-law. Mean distribution of gaze step length (left plot) and of Kolmogorov-Smirnov Distance (KSD; right plot) across all subjects (grey) and within static (blue) and dynamic (red) viewers separately. Interestingly, the lowest KSD values (i.e., indicating a distribution closer to a power-law) are more frequent for static viewers than dynamic viewers, while the opposite pattern can be noticed for the upper tail. This indicates that on average Static Viewers show a more power-law-like distribution of gaze steps length than Dynamic Viewers.

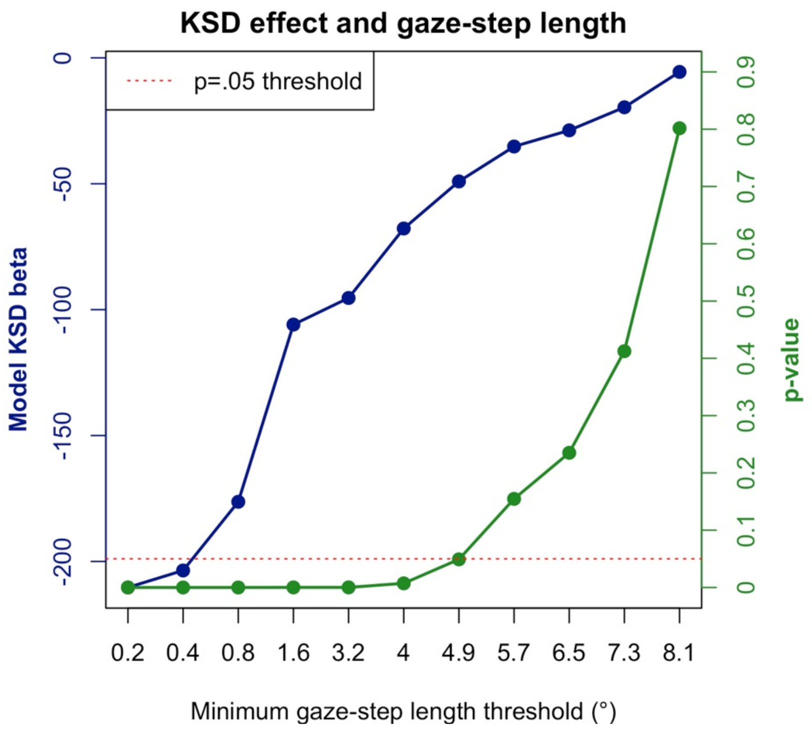

Supplementary Figure 8. Control analysis on the reliability of the effect of KSD on PC1 across different gaze step lengths. To test if our results were driven by eye-tracker's spatial accuracy, we repeated our linear regression model to predict PC1 values (N=114) by iteratively computing KSD distance between gaze steps and power law distribution on different subset of trials. Specifically, we iteratively removed gaze-steps shorter than a threshold varying from 0.2° to 8.1°. The figure shows that the KSD effect on PC1 remained significant across many thresholds (0.2°-4.9°) well within the accuracy of the eye-tracker. The fact that the relationship PC1-KSD is not any longer significant after removing all gaze-steps <4.9° suggests that power-law dynamics could not explain saccades behavior, rather intrinsic dynamics might be more critical for fixational eye movements.

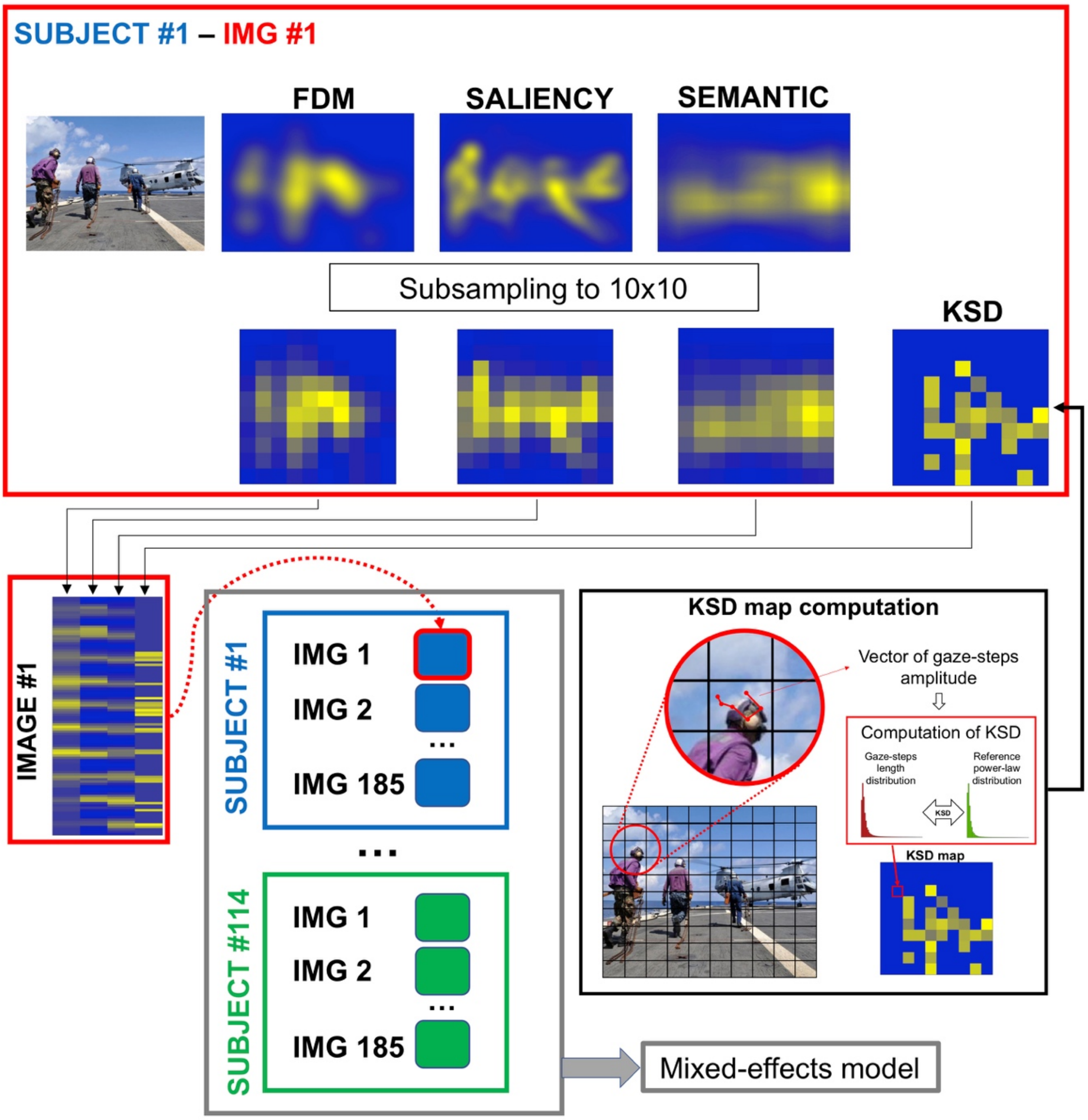

Supplementary Figure 9. Graphical representation of the method used to build the fixation topography models. To compute KSD maps (Black box) we first subsampled the image space (683x512 pixels) to a 10x10 matrix. Then, for each cell we extracted the vector of gaze-steps length and calculated a value of KSD indicating the distance to a power law distribution (see Methods section for details). Then (red boxes), we subsampled FDM, saliency and semantic maps to match the dimensions of the N=185 KSD map (10x10), which were then vectorized and concatenated in a dataframe (grey box) which was then used for the subsequent mixed-effect models comparison. KSD=Kolmogorov-Smirnov Distance; FDM=Fixation Density Map.

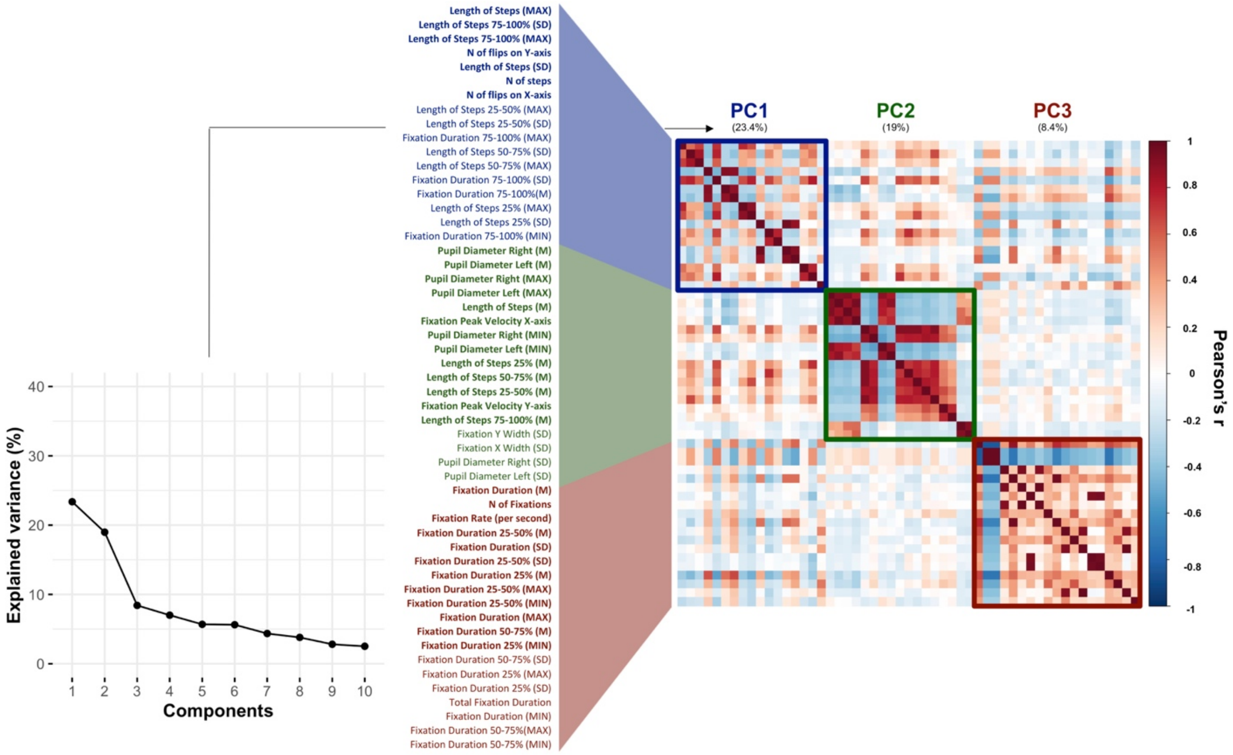

Supplementary Figure 10. Correlation matrix of visual exploration features in blank screen viewing. The line plot shows the variance explained by different principal components (PCs). The matrix shows the correlation (Pearson's r) between features, which are ordered according to their loadings in the first three PCs. The color of Y axis labels indicates the PC with the highest loading for the corresponding feature, and features written in bold are those with loadings > 0.2.

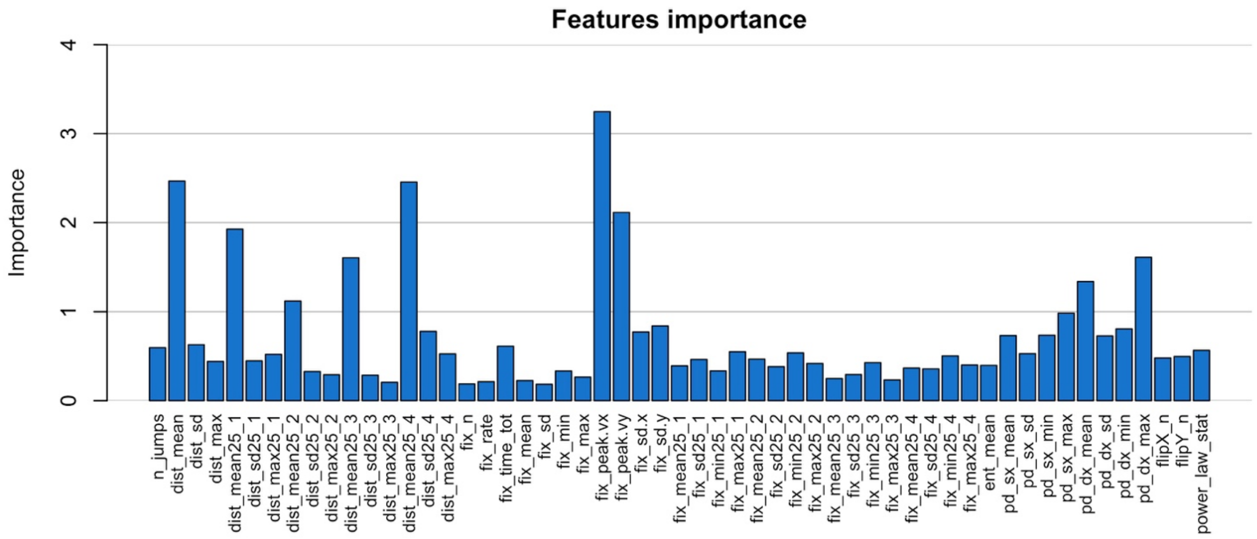

Supplementary Figure 11. Feature importance in Random Forest model. A description of each feature can be found in Supplementary Table 2.

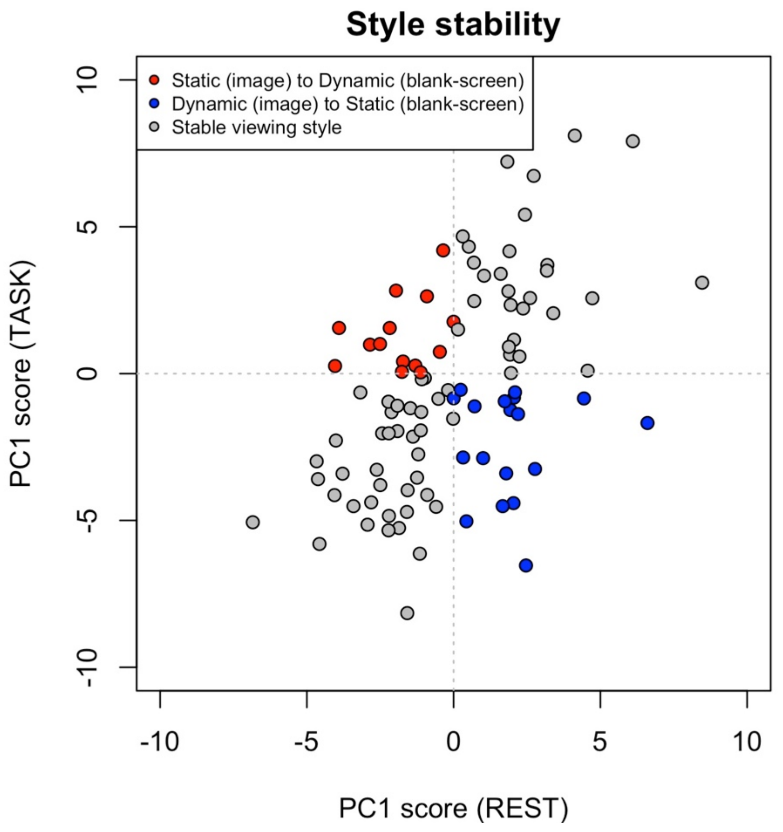

Supplementary Figure 12. Stability of viewing style between conditions. We checked the stability of the viewing style between image-viewing and blank screen viewing conditions. To this end, we applied PC1 loadings derived from the image-viewing condition to features computed in the blank-screen viewing condition (excluding 14 subjects for the reasons explained in the methods section). This procedure allowed us to compare PC1 scores obtained in different conditions, within the same component space. It should be noted that positive PC1 scores indicate Static viewing style, while negative values indicate Dynamic viewing style. The figure below shows the correlation between PC1 scores computed from image-viewing ('TASK') features (Y axis) and those computed from blank-screen viewing ('REST') condition (X axis). Blue dots represent Dynamic viewers who switched to Static in the blank-screen viewing (18%). Red dots depict subjects showing the opposite path (14%). Grey dots represent subjects whose viewing style was stable across conditions (68%).

Supplementary Tables

Category	What is measured	Variable	Sample (n=114) Mean (SD)
Demographic		Age	23.5 (2.45)
		Sex	67 F
		Years of education	16.6 (1.27)
Cognitive	Impulsivity	Stroop Test Words – time (s)	12.35 (1.94)
		Stroop Test Words – errors	0.04 (0.21)
		Stroop Test Colors – time (s)	15.46 (2.1)
		Stroop Test Colors – errors	0.52 (0.72)
		Stroop Test Interference - errors	0.18 (0.75)
		Stroop Test Interference – time (s)	9.53 (3.64)
	Verbal Memory	Digit Span Forward	6.42 (1.08)
		Digit Span Backward	4.89 (1.28)
	Visuospatial ability	Rey-Osterrieth Figure - Copy	35.49 (1.03)
	Visuospatial Memory	Rey-Osterrieth Figure – Recall	26.38 (4.78)
Personality	Big-Five	NEO-FFI - Neuroticism	25.33 (9.14)
		NEO-FFI - Extraversion	28.4 (6.83)
		NEO-FFI - Openness	34.14 (6.01)
		NEO-FFI - Agreeableness	30.63 (5.99)
		NEO-FFI - Conscientiousness	29.89 (8.1)
Behavioural State	Anxiety	DASS - Anxiety	5.58 (4.21)
	Depression	DASS - Depression	7.21 (4.86)
	Stress	DASS - Stress	9.65 (4.49)
Behavioural traits	Behavioural Impulsivity	BIS	22.76 (4.67)
		BAS – Reward Responsiveness	21.04 (3.18)
		BAS - Drive	12.41 (3.11)
		BAS – Fun Seeking	13.03 (3.29)

Supplementary Table 1. Sample demographics and behavioral measures.

Features	Code	Description
N of Fixations	fix_n	Number of fixations
Fixation Rate (per second)	fix_rate	Number of fixations per second on average
Fixation Duration (M)	fix_mean	Mean fixations' duration (i.e., mean interval between consecutive saccades)
Fixation Duration (SD)	fix_sd	Standard deviation of fixations' duration
Fixation Duration (MIN)	fix_min	Minimum fixation duration
Fixation Duration (MAX)	fix_max	Maximum fixation duration
Fixation Duration 25% (M)	fix_mean25_1	Mean duration of the first 25% of fixations timeseries
Fixation Duration 25% (SD)	fix_sd25_1	Standard deviation of fixations duration in the first quarter visual exploration
Fixation Duration 25% (MIN)	fix_min25_1	Minimum fixation duration in the first quarter visual exploration
Fixation Duration 25% (MAX)	fix_max25_1	Maximum fixation duration in the first quarter visual exploration
Fixation Duration 25-50% (M)	fix_mean25_2	Mean duration of the fixations from 25% to 50%
Fixation Duration 25-50% (SD)	fix_sd25_2	Standard deviation of fixations duration in the second quarter of visual exploration
Fixation Duration 25-50% (MIN)	fix_min25_2	Minimum fixation duration in the second quarter visual exploration
Fixation Duration 25-50% (MAX)	fix_max25_2	Maximum fixation duration in the second quarter visual exploration
Fixation Duration 50-75% (M)	fix_mean25_3	Mean fixations duration in the third quarter of visual exploration
Fixation Duration 50-75% (SD)	fix_sd25_3	Standard deviation of fixations duration in the third quarter visual exploration
Fixation Duration 50-75% (MIN)	fix_min25_3	Minimum fixation duration in the third quarter visual exploration
Fixation Duration 50-75%(MAX)	fix_max25_3	Maximum fixation duration in the third quarter visual exploration
Fixation Duration 75-100%(M)	fix_mean25_4	Mean fixation duration in the last quarter of the visual exploration
Fixation Duration 75-100% (SD)	fix_sd25_4	Standard deviation of fixations duration in the last quarter visual exploration
Fixation Duration 75-100% (MIN)	fix_min25_4	Minimum fixation duration in the last quarter of visual exploration
Fixation Duration 75-100% (MAX)	fix_max25_4	Maximum fixation duration in the last quarter visual exploration
Total Fixation Duration	fix_time_tot	Global time spent in fixations
Fixation Y Width (SD)	fix_sd.x	Standard deviation of fixations width on the Y axis
Fixation X Width (SD)	fix_sd.y	Standard deviation of fixations width on the X axis
Fixation Peak Velocity X-axis	fix_peak.vx	Fixation peak velocity on X axis
Fixation Peak Velocity Y-axis	fix_peak.vy	Fixation peak velocity on Y axis
Pupil Diameter Right (M)	pd_dx_mean	Mean of right pupil diameter (in mm)
Pupil Diameter Right (SD)	pd_dx_sd	Standard deviation of right pupil diameter (in mm)
Pupil Diameter Right (MIN)	pd_dx_min	Minimum of right pupil diameter (in mm)
Pupil Diameter Right (MAX)	pd_dx_max	Maximum of right pupil diameter (in mm)
Pupil Diameter Left (M)	pd_sx_mean	Mean of left pupil diameter (in mm)
Pupil Diameter Left (SD)	pd_sx_sd	Standard deviation of left pupil diameter (in mm)
Pupil Diameter Left (MIN)	pd_sx_min	Minimum of left pupil diameter (in mm)
Pupil Diameter Left (MAX)	pd_sx_max	Maximum of left pupil diameter (in mm)
Exploration Time (TOT)	time_exp_tot	Total exploration time
Exploration Time (M)	time_exp_mean	Mean image exploration time
Exploration Time (SD)	time_exp_sd	Standard deviation of images exploration time
Exploration Time (MIN)	time_exp_min	Minimum image exploration time
Exploration Time (MAX)	time_exp_max	Maximum image exploration time
N of flips on Y-axis	flipX_n	Number of flips (i.e., change of direction) on Y-axis
N of flips on X-axis	flipY_n	Number of flips (i.e., change of direction) on X-axis
N of steps	n_jumps	Number of gaze steps
Length of Steps (M)	dist_mean	Mean of gaze steps length
Length of Steps (SD)	dist_sd	Standard deviation of the gaze steps length
Length of Steps (MAX)	dist_max	Maximum gaze steps length
Length of Steps 25% (M)	dist_mean25_1	Mean of the gaze steps length in the first quarter visual exploration
Length of Steps 25% (SD)	dist_sd25_1	Standard deviation of gaze steps length in the first quarter visual exploration
Length of Steps 25% (MAX)	dist_max25_1	Maximum gaze steps length in the first quarter visual exploration
Length of Steps 25-50% (M)	dist_mean25_2	Mean of the gaze steps length in the second quarter visual exploration
Length of Steps 25-50% (SD)	dist_sd25_2	Standard deviation of gaze steps length in the second quarter visual exploration
Length of Steps 25-50% (MAX)	dist_max25_2	Maximum gaze steps length in the second quarter visual exploration
Length of Steps 50-75% (M)	dist_mean25_3	Mean of the gaze steps length in the third quarter visual exploration
Length of Steps 50-75% (SD)	dist_sd25_3	Standard deviation of gaze steps length in the third quarter visual exploration
Length of Steps 50-75% (MAX)	dist_max25_3	Maximum gaze steps length in the third quarter visual exploration
Length of Steps 75-100% (M)	dist_mean25_4	Maximum gaze steps length in the last quarter visual exploration
Length of Steps 75-100% (SD)	dist_sd25_4	Standard deviation of gaze steps length in the last quarter visual exploration
Length of Steps 75-100% (MAX)	dist_max25_4	Maximum gaze steps length in the last quarter of visual exploration
**Features used in the linear regression model**		
Saliency info in fixations (M)	fix_SAL_mean (SAL)	Mean quantity of saliency information collected in fixations
Semantic info in fixations (M)	fix_SEM_mean (SEM)	Mean quantity of semantic information collected in fixations
Entropy of visual exploration (M)	ent_mean (ShEn)	Mean Shannon entropy value calculated on the visual exploration pattern
Power law similarity of gaze	power_law_stat (KSD)	Distance between empirical gaze steps distribution and a reference power law computed by means of the Kolmogorov-Smirnov test (i.e., Kolmogorov-Smirnov Distance, KSD)

Supplementary Table 2. Description of the eye movements features extracted.

Features	Image-viewing				Blank screen viewing		
	PC1	PC2	PC3		PC1	PC2	PC3
Fixation Duration 25% (M)	**0.241**	-0.031	0.068		0.05	-0.02	**0.218**
Fixation Duration (M)	**0.241**	-0.002	0.052		-0.047	0.002	**0.293**
Fixation Duration 25-50% (M)	**0.24**	-0.003	0.059		0.103	-0.042	**0.262**
Fixation Duration (SD)	**0.232**	0.027	0.051		-0.051	-0.003	**0.249**
Fixation Duration 50-75% (M)	**0.232**	0.003	0.057		-0.004	-0.003	**0.211**
Fixation Duration 25-50% (SD)	**0.226**	0.004	0.051		0.103	-0.055	**0.239**
Fixation Duration 75-100%(M)	**0.216**	0.021	0.018		-0.15	0.04	0.078
Fixation Duration 50-75% (SD)	**0.214**	0.029	0.044		0.012	0.007	0.189
Fixation Duration 25% (SD)	**0.213**	-0.013	0.081		0.063	-0.013	0.185
Fixation Y Width (SD)	0.189	-0.022	-0.059		0.045	0.177	0.043
Fixation Duration 75-100% (SD)	0.188	0.069	0.015		-0.152	0.041	0.044
Fixation X Width (SD)	0.164	-0.02	-0.046		-0.012	0.176	0.014
Fixation Peak Velocity Y-axis	0.14	0.016	-0.144		0.102	**0.228**	0.079
Fixation Duration 25-50% (MAX)	0.127	0.102	0.044		0.103	-0.054	**0.217**
Fixation Duration 25% (MAX)	0.108	0.063	0.063		0.065	-0.012	0.187
Fixation Duration 50-75% (MIN)	0.048	0.043	-0.005		-0.059	-0.026	0.108
Fixation Duration 25-50% (MIN)	0.039	-0.012	0.022		0.008	0.035	**0.213**
Fixation Duration (MIN)	0.028	-0.008	-0.009		-0.073	0.071	0.177
Pupil Diameter Left (M)	-0.184	0.02	-0.003		0.179	**-0.266**	0.046
Pupil Diameter Right (M)	-0.171	0.032	0.037		0.155	**-0.27**	0.057
Pupil Diameter Left (SD)	-0.168	-0.005	-0.052		0.032	-0.12	-0.052
Pupil Diameter Left (MAX)	-0.165	0.053	-0.023		0.169	**-0.257**	0.021
Pupil Diameter Right (SD)	-0.155	0.002	-0.005		0.028	-0.135	-0.047
Pupil Diameter Right (MIN)	-0.141	-0.012	0.054		0.111	**-0.252**	0.047
Pupil Diameter Right (MAX)	-0.134	0.045	0.039		0.154	**-0.266**	0.05
Pupil Diameter Left (MIN)	-0.131	-0.033	0.046		0.14	**-0.245**	0.067
Exploration Time (M)	-0.018	**0.32**	0.004				
N of flips on Y-axis	0.003	**0.315**	0.038		**-0.251**	0.071	0.017
N of flips on X-axis	0.005	**0.314**	0.036		**-0.219**	0.081	0.068
N of steps	0.006	**0.31**	0.044		**-0.236**	0.123	-0.022
Total Fixation Duration	0.044	**0.299**	0.032		-0.151	0.051	0.178
N of Fixations	-0.155	**0.289**	-0.015		0.044	0.009	**-0.283**
Exploration Time (TOT)	-0.02	**0.285**	-0.029				
Exploration Time (MIN)	-0.022	**0.252**	0.022				
Fixation Duration (MAX)	0.082	0.197	0.02		-0.066	0.01	**0.211**
Fixation Duration 75-100% (MAX)	0.064	0.196	0.014		-0.171	0.062	0.017
Fixation Rate (per second)	-0.187	0.193	-0.008		0.044	0.009	**-0.283**
Fixation Duration 50-75%(MAX)	0.113	0.131	0.027		0.003	0.012	0.169
Exploration Time (MAX)	0.01	0.118	0.081				
Exploration Time (SD)	0.047	-0.194	-0.001				
Fixation Duration 25% (MIN)	0.076	-0.082	0.037		-0.01	0.016	**0.206**
Length of Steps (SD)	-0.091	-0.042	**-0.333**		**0.246**	0.109	-0.053
Length of Steps 25% (SD)	-0.114	-0.021	**-0.313**		0.141	0.111	-0.063
Length of Steps 50-75% (SD)	-0.077	-0.078	**-0.305**		0.159	0.116	-0.074
Length of Steps 25-50% (SD)	-0.077	-0.103	**-0.303**		0.19	0.066	-0.086
Length of Steps 75-100% (SD)	-0.06	0.077	**-0.251**		**0.259**	0.07	0.02
Length of Steps (M)	0.105	-0.019	**-0.233**		0.133	**0.254**	0.002
Length of Steps 25% (M)	0.081	-0.004	**-0.232**		0.096	**0.242**	-0.019
Length of Steps 50-75% (M)	0.1	-0.056	**-0.221**		0.121	**0.23**	0.006
Length of Steps 25-50% (M)	0.097	-0.065	**-0.221**		0.114	**0.229**	-0.021
Length of Steps 50-75% (MAX)	-0.033	0.061	**-0.221**		0.155	0.057	-0.018
Length of Steps (MAX)	-0.058	0.095	**-0.217**		**0.263**	0.007	0.058
Length of Steps 25% (MAX)	-0.052	0.095	**-0.216**		0.148	0.072	0.05
Length of Steps 25-50% (MAX)	-0.025	0.027	-0.19		0.195	0.013	-0.024
Length of Steps 75-100% (M)	0.107	0.056	-0.189		0.157	**0.222**	0.046
Length of Steps 75-100% (MAX)	-0.03	0.107	-0.177		**0.256**	0.021	0.092
Fixation Peak Velocity X-axis	0.119	-0.01	-0.156		0.069	**0.253**	0.058
Fixation Duration 75-100% (MIN)	0.009	0.001	0.026		-0.086	0.04	0.075

Supplementary Table 3. Principal Components Analysis loadings. Matrix of rotated loadings obtained in the Principal Component Analysis run on the image-viewing data and on blank screen viewing data. Values in bold are those with absolute value higher than the chosen threshold= 0.2.

Model comparison type	Model	R^2	Predictors	AIC	BIC	F or χ^2, [df]	p-value
Spatiotemporal features	M1	.083	SAL	637.7	645.9	-	-
	M2	.087	SAL + SEM	638.3	649.2	1.66 [1,111]	.2
	M3	.099	SAL + SEM + ShEn	637.7	651.4	2.81 [1,110]	.097
	**M4**	**.198**	**SAL + SEM + ShEn + KSD**	**625.5**	**641.9**	**14.49 [1,109]**	**<.001***
Fixation topography	MM1	.151	SAL	-87011	-86958	-	-
	MM2	.195	SAL + SEM	-102168	-102104	15158.6 [1]	<.001*
	**MM3**	**.196**	**SAL + SEM + KSD**	**-102847**	**-102772**	**681.1 [1]**	**<.001***

Supplementary Table 4. Likelihood Ratio Tests (LRTs) results. The table shows the results of two model comparisons ran by means of LRTs on two sets of models. The first set of linear regression models (M1 to M4) used saliency, semantic, Shannon entropy, and KSD to predict PC1 scores. The second set of models used saliency, semantic and KSD to predict fixation topography (image-specific Fixation Density Maps, FDMs). For each model Akaike Information Criterion (AIC), Bayesian Information Criterion (BIC), statistic value (F for linear regression, χ^2 for mixed-effects models) and p-values are reported. SAL = saliency information; SEM=semantic information; ShEn = Shannon Entropy of visual exploration topography; KSD = Kolmogorov-Smirnov Distance. *=p-value below .05. Best models are noted in bold.

Model type	Predictor	Beta	SE	t	p
Spatiotemporal features	Saliency information in fixations (SAL)	-32.03	41.17	-.78	.44
	Semantic information in fixations (SEM)	-29.59	33.78	-.88	.38
	Shannon Entropy (ShEn)	1.71	.97	1.76	.08
	**Kolmogorov-Smirnov Distance (KSD)**	-151.78	40.06	-3.79	<.001 *
Fixation topography model	**Saliency information in fixations (SAL)**	.37	.0022	167.16	<.001*
	**Semantic information in fixations (SEM)**	.24	.0019	123.32	<.001*
	**Kolmogorov-Smirnov Distance (KSD)**	-.21	.0079	-26.11	<.001*
Cognitive-Personality model	Sex	.97	.98	.99	.32
	**Age**	.67	.25	2.66	.01 *
	Education	.21	.39	.54	.59
	**Stroop (Time)**	-.29	.12	-2.36	.02 *
	Span Forward	-.70	.43	-1.60	.11
	Span Backward	.30	.34	.90	.37
	Rey-Osterrieth figure – Delayed Recall	.10	.09	1.09	.28
	NEO-FFI - Neuroticism	.01	.07	.10	.92
	NEO-FFI – Extraversion	.03	.09	.29	.77
	NEO-FFI – Openness	.14	.07	1.93	.06 (*)
	NEO-FFI – Agreableness	-.05	.09	-.63	.53
	NEO-FFI – Coscientiousness	-.01	.07	-.12	.90
	BIS	-.02	.12	-.18	.86
	BAS – Reward Responsiveness	.09	.15	.63	.53
	BAS – Drive	.12	.20	.57	.57
	BAS – Fun Seeking	-.09	.15	-.61	.54

Supplementary Table 5. Statistics for the linear regression models. Beta, Standard Error (SE), t and p-value are reported for each predictor. The set of predictors included in the Cognitive-Personality model resulted from a stepwise procedure. *=p-value below .05; (*)=trend to significance (i.e., p-value between .05 and .1). Significant predictors are written in bold.
