# Supplementary material for: Visual exploration dynamics are low-dimensional and driven by intrinsic factors
Source: Commun Biol. 2021 Sep 17;4:1100. doi: 10.1038/s42003-021-02608-x (PMC8448835; doi:10.1038/s42003-021-02608-x)
Supplement: Supplementary file 2 — Reporting Summary [file 42003_2021_2608_MOESM2_ESM.pdf]

nature research

Corresponding author(s): Maurizio Corbetta

Last updated by author(s): Jul 20, 2021

Reporting Summary

Nature Research wishes to improve the reproducibility of the work that we publish. This form provides structure for consistency and transparency in reporting. For further information on Nature Research policies, see our Editorial Policies and the Editorial Policy Checklist.

Statistics

For all statistical analyses, confirm that the following items are present in the figure legend, table legend, main text, or Methods section.

n/a	Confirmed	
☐	☒	The exact sample size (*n*) for each experimental group/condition, given as a discrete number and unit of measurement
☐	☒	A statement on whether measurements were taken from distinct samples or whether the same sample was measured repeatedly
☐	☒	The statistical test(s) used AND whether they are one- or two-sided<br>*Only common tests should be described solely by name; describe more complex techniques in the Methods section.*
☐	☒	A description of all covariates tested
☐	☒	A description of any assumptions or corrections, such as tests of normality and adjustment for multiple comparisons
☐	☒	A full description of the statistical parameters including central tendency (e.g. means) or other basic estimates (e.g. regression coefficient) AND variation (e.g. standard deviation) or associated estimates of uncertainty (e.g. confidence intervals)
☐	☒	For null hypothesis testing, the test statistic (e.g. *F*, *t*, *r*) with confidence intervals, effect sizes, degrees of freedom and *P* value noted<br>*Give P values as exact values whenever suitable.*
☒	☐	For Bayesian analysis, information on the choice of priors and Markov chain Monte Carlo settings
☒	☐	For hierarchical and complex designs, identification of the appropriate level for tests and full reporting of outcomes
☐	☒	Estimates of effect sizes (e.g. Cohen's *d*, Pearson's *r*), indicating how they were calculated

Our web collection on statistics for biologists contains articles on many of the points above.

Software and code

Policy information about availability of computer code

Data collection	Eye-movement data were collected through a Tobii T120 eye-tracker, by means of a custom E-Prime 2 script.
Data analysis	Data were analyzed by means of custom R (version 3.6.2), Matlab (version R2017b) and Python (version 3.7) code.

For manuscripts utilizing custom algorithms or software that are central to the research but not yet described in published literature, software must be made available to editors and reviewers. We strongly encourage code deposition in a community repository (e.g. GitHub). See the Nature Research guidelines for submitting code & software for further information.

Data

Policy information about availability of data

All manuscripts must include a data availability statement. This statement should provide the following information, where applicable:

- Accession codes, unique identifiers, or web links for publicly available datasets
- A list of figures that have associated raw data
- A description of any restrictions on data availability

The data that support the findings of this study are available from the corresponding author on reasonable request. The source data used for the main figures are available at https://osf.io/2rkx9/.

Field-specific reporting

Behavioural & social sciences study design

All studies must disclose on these points even when the disclosure is negative.

Study description	The design of the study is cross-sectional, with collection of eye-movement and cognitive (i.e., tests) data.
Research sample	We recruited N=120 participants (19-34 y.o., mean age 23.4, SD=2.42; 49M) among students of the University of Padua or citizens. Then, 6 participants were excluded from the analysis for excessive missing data (N=5) or for color-blindness (N=1). The final sample was thus composed of N=114 participants (mean age=23.5, SD=2.45, 67 F). Since we aimed at studying inter-individual variability in normal eye-movements patterns during free-viewing, we decided to focus only on healthy young participants to rule out the possibility of biased data due to aging or age-related diseases.
Sampling strategy	The sampling procedure was random. We decided to recruit a final sample of N≥100 participants to have a reasonably large sample to study inter-individual variability in eye-movements. In our opinion, this sample size is sufficient to highlight the presence of clusters in a data-driven fashion.
Data collection	Data were collected by means of an eyetracker (Tobii T120), paper and pencil cognitive tests (i.e., Stroop Test, Digit Span, Rey-Osterrieth Complex Figure), and questionnaires (i.e., NEO-FFI, DASS, BIS-BAS) which were sent by e-mail after the session. During the data collection only 1 researcher was present with the participant. No between-subjects experimental conditions were involved.
Timing	Data collection took place between January 31st 2018 and June 5th 2018.
Data exclusions	Six participants were excluded from the analyses after data collection for the following reasons: excessive data loss, defined as less than 50% of usable data in more than 25% of trials (N=3), interruption of the experimental session due to panic attack (N=1) or eyes irritation (N=1), and colour-blindness revealed after the experimental session was completed (N=1).
Non-participation	No participants dropped out.
Randomization	Participants were not allocated into experimental groups.

Reporting for specific materials, systems and methods

We require information from authors about some types of materials, experimental systems and methods used in many studies. Here, indicate whether each material, system or method listed is relevant to your study. If you are not sure if a list item applies to your research, read the appropriate section before selecting a response.

Materials & experimental systems

n/a	Involved in the study	
☒	☐	Antibodies
☒	☐	Eukaryotic cell lines
☒	☐	Palaeontology and archaeology
☒	☐	Animals and other organisms
☐	☒	Human research participants
☒	☐	Clinical data
☒	☐	Dual use research of concern

Methods

n/a	Involved in the study	
☒	☐	ChIP-seq
☒	☐	Flow cytometry
☒	☐	MRI-based neuroimaging

Human research participants

Policy information about studies involving human research participants

Population characteristics	See above.
Recruitment	Participants were recruited using social media (Facebook) and face to face direct contact.
Ethics oversight	Ethical Committee for the Psychological Research of the University of Padova.

Note that full information on the approval of the study protocol must also be provided in the manuscript.
